# Separase Inhibition Enhances Gefitinib Sensitivity of Lung Cancer via PTBP1/TAK1/RIPK1‐Mediated PANoptosis

**DOI:** 10.1002/mco2.70432

**Published:** 2025-10-20

**Authors:** Zhouyangfan Peng, Liangpeng Xie, Sufang Zhou, Yapei Li

**Affiliations:** ^1^ Health Management Center The Third Xiangya Hospital Central South University Changsha China; ^2^ School of Basic Medical Sciences Guangxi Medical University Nanning China

**Keywords:** gefitinib, lung cancer, PANoptosis, RIPK1, separase, TAK1

## Abstract

Gefitinib is the most frequently employed anti‐lung cancer drug, but its clinical effectiveness is often compromised due to the development of drug resistance. Given that gefitinib failure to long‐term inhibition of the growth of lung carcinoma cell lines, which mirrors the resistance observed in clinical patients, new approaches to improve the curative effect of gefitinib should be found. Surprisedly, inhibiting separase with the specific inhibitor Sepin‐1 has been found to effectively enhance gefitinib‐induced cytotoxic in lung cancer cell by promoting the development of PANoptosis, which includes pyroptosis, apoptosis, and necroptosis. Moreover, in vivo experiments also demonstrated that the combination of Sepin‐1 and gefitinib can induce significant regression of lung xenograft tissues. Mechanically, loss of separase plus gefitinib decreases the expression of PTBP1 and TAK1. Overexpression of PTBP1 or TAK1 suppresses this interaction‐induced PANoptosis by promoting the inactivation of RIPK1. In addition, clinical data showed that better effective of gefitinib maybe associated with lower separase expression or higher PANoptosis marker expression in patient lung carcinoma tissues. Thus, these findings provide a novel anti‐lung cancer strategy and highlight separase as a potential target for overcoming gefitinib resistance in lung cancer treatment.

## Graphical abstract

Inhibiting separase with the specific inhibitor Sepin‐1 has been found to effectively enhance gefitinib‐induced cytotoxic in lung cancer cell. Mechanically, separase inhibitor Sepin‐1 plus gefitinib decreases the expression of PTBP1 and TAK1 to trigger RIPK1‐dependent PANoptosis.

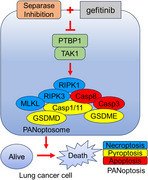

## 1 Introduction

Lung cancer remains the leading cause of cancer‐related mortality globally, with approximately 2.2 million new cases and 1.8 million death cases annually [1]. A significant factor contributing to the development and progression of this disease is the abnormal mutation of the epidermal growth factor receptor (EGFR) gene [2].

Gefitinib, an EGF receptor tyrosine kinase inhibitors (EGFR‐TKIs), is currently the first‐line treatment for advanced lung cancer patients with EGFR mutation. However, the efficacy of gefitinib is often compromised by primary or acquired resistance [2, 3]. Primary gefitinib resistance is mainly due to KRAS mutation and site‐specific mutation resulting in varying sensitivity levels [4]. In addition, approximately 65% of lung cancer patients eventually develop acquired gefitinib resistance after several months of treatment. Secondary EGFR mutation of T790M and C797S, BRAF and PIK3CA‐induced EGFR signaling pathway hyperactivation, bypass activation, and cell phenotype transformation are major factors contributing to acquired gefitinib resistance [5, 6]. Additionally, recent studies confirmed the role of epigenetic regulation in gefitinib resistance. For instance, KIAA1429 has been shown to promote tumorigenesis and gefitinib resistance in lung adenocarcinoma by activating the JNK/MAPK pathway in an m6A‐RNA methylation‐dependent manner [3]. Moreover, autophagy‐related mechanisms are also implicated in the development of resistance to EGFR‐TKIs. Autophagy also has been found to enhance the resistance of lung cancer cells to gefitinib [7]. Therefore, overcoming gefitinib resistance is crucial for improving treatment outcomes in lung cancer patients with EGER mutation.

Gefitinib inhibits the growth of lung cancer cells by inducing apoptosis. However, our results showed that gefitinib‐triggered apoptosis only temporarily inhibits lung cancer cell growth due to the occurrence of apoptosis resistance. Therefore, activation of other death pathways in lung cancer cells may effectively overcome this resistance. Different forms of programmed cell death, playing a crucial role in various pathological and physiological processes, is mediated by distinct executor [8]. Following the discovery of caspase‐8/3‐mediated apoptosis as the earliest form of programmed cell death, other types, such as receptor interacting protein kinase 3 (RIPK3)/ mixed lineage kinase domain‐like (MLKL)‐mediated necroptosis and gasdermin (GSDMD)/gasdermin E (GSDME)‐mediated pyroptosis, have also been discovered [9, 10]. But recent research has found that the occurrence of individual forms of programmed cell death often coincides with the presence of other types, which is regulated by common targets [11, 12]. Based on this discovery, PANoptosis, involved in apoptosis, necroptosis, and pyroptosis, has been proposed [13]. Current research has confirmed that receptor interacting protein kinase 1 (RIPK1) is a crucial executor to induce PANoptosis [14]. Activated RIPK1 recruits fas‐associated death domain (FADD) proteins to activate caspase‐8/caspase‐3 signaling, resulting in cell apoptosis. Activated caspase‐8 and caspase‐3 further cleave GSDMD and GSDME to mediate pyroptosis. In this process, while caspase‐8 is excessively consumed, RIPK1 shifts to trigger the phosphorylation and activation of RIPK3 and MLKL to cause necroptosis [15, 16]. Thus, we intend to induce PANoptosis through combined treatment.

Separase is a cysteine protease that plays a pivotal role in promoting the separation of sister chromatids during cell mitosis [17]. It acts as a speed regulator in the process of cell mitosis and serves as a trigger factor for the transition at the end of cell division in all eukaryotic cells [18]. Separase is considered as an oncogene, since it is overexpressed in various human cancers such as breast cancer, osteosarcoma, and prostate cancer [19]. Overexpression of separase in mice has been identified to elicit the formation of adenocarcinomas [20, 21]. Nevertheless, the role of separase in lung cancer is still unknown.

Our clinical analysis identified that separase is highly expressed in lung carcinoma patients. Patients with high expression of separase showed an increased risk of lung cancer‐related death. However, inhibiting separase expression alone fails to inhibit the growth of lung cancer cells. A possible reason attributed to this result is that clinical patients are undergoing other drug treatments at the same time. Hence, we explored whether inhibiting the expression of separase could enhance the sensitivity of other anti‐cancer drugs, and our results indicated that separase inhibition plays a novel role to enhance gefitinib sensitivity in lung cancer cells by inducing PANoptosis.

## 2 Results

### 2.1 Gefitinib Temporarily Inhibits the Growth of Lung Cancer Cells

**FIGURE 1:**
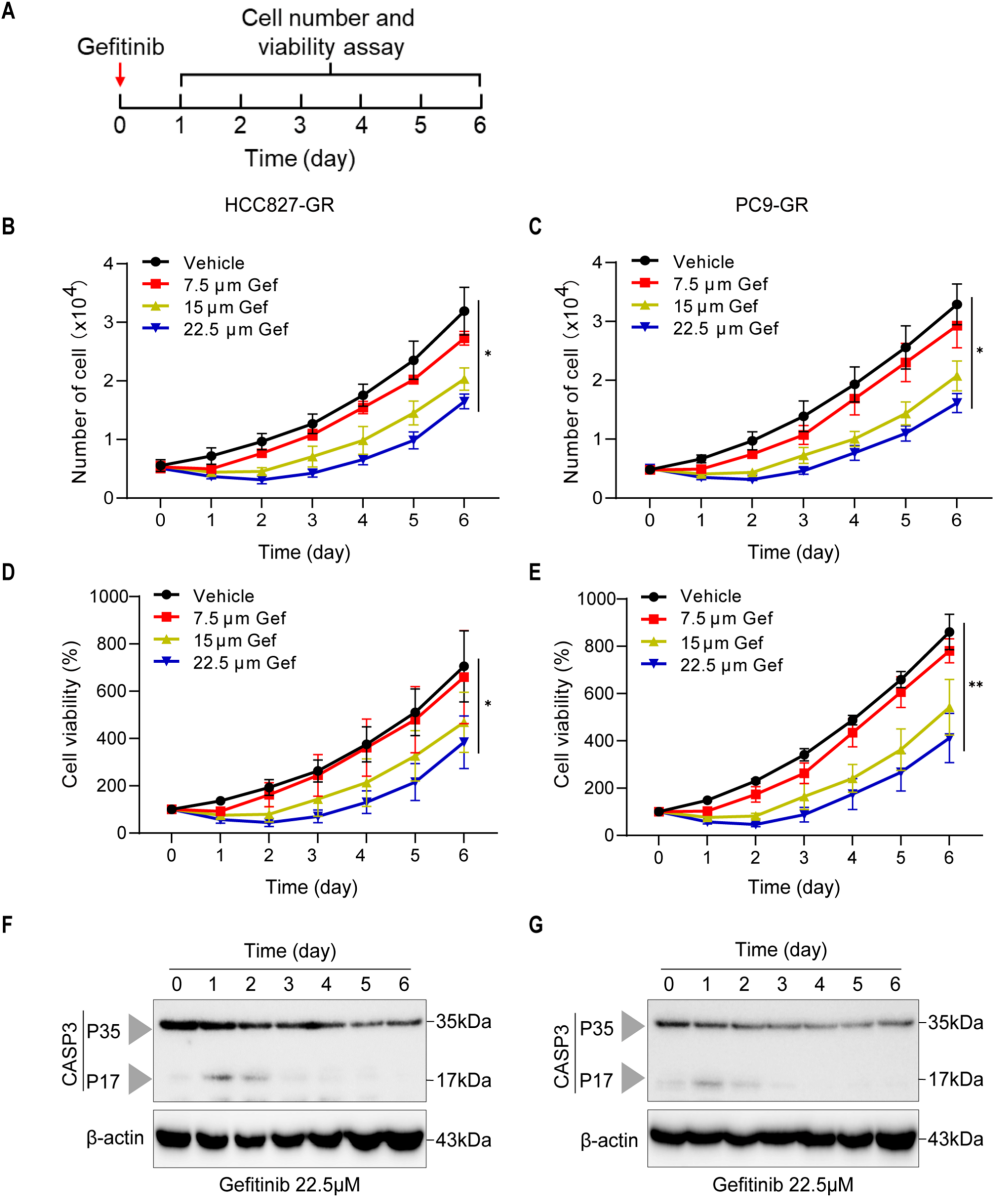
The proliferation of lung cancer cells cannot be sustainedly inhibited by using gefitinib alone. Gefitinib fails to inhibit the proliferative potential of human lung cancer cells. (A) Drug‐stimulating strategy: human lung cancer cells were stimulated with gefitinib for 7 days. The cell number and viability were measured daily throughout the experiment. (B and C) The residual number of the HCC827‐GR (B) and PC9‐GR (C) cells treated with gefitinib (7.5, 15, and 22.5 µM) for 7 days (*n* = 3). (D and E) The cell viability of HCC827‐GR (D) and PC9‐GR (E) cells stimulated with gefitinib (7.5, 15, and 22.5 µM) for 7 days was measured using CCK‐8 assays. (F and G) Immunoblot analysis of pro‐caspase‐3 and cleaved caspase‐3 in HCC827‐GR (F) and PC9‐GR (G) cell lines stimulated with gefitinib (22.5 µM) for 7 days. β‐Actin was used as the internal control (*n* = 3). (B–E) Data are shown as mean ± SD (*p < 0.05, **p < 0.01, ***p < 0.001, two‐tailed *t*‐test). (F and G) Data are representative of at least three independent experiments.

The gefitinib‐resistant lung cancer cell HCC827 (HCC827‐GR) and PC9 (PC9‐GR) were selected to evaluate the cytotoxicity of gefitinib. HCC827‐GR cells carry the T790M mutation in the EGFR kinase domain, which increases ATP affinity and diminishes gefitinib's effectiveness. In addition, PC9‐GR cells not only possess the T790M mutation but also activate compensatory pathways, such as MET amplification, further enhancing the resistance profile [22, 23]. To investigate whether high doses of gefitinib can sustainably inhibit the proliferation of these lung cancer cells, HCC827‐GR andPC9‐GR cells were stimulated with 7.5, 15, and 22.5 µM gefitinib for 7 days to observe the long‐term pharmacological effects (Figure 1A). We observed a concentration‐dependent decrease in cell viability within 48 h after gefitinib stimulation, but this decrease recovered after 72 h (Figure 1B–,E). Apoptosis resistance is a major reason contributing to the failure of gefitinib treatment. To confirm whether high doses of gefitinib still induces apoptosis resistance after long‐term stimulation, we performed western blotting and found that the cleavage of apoptosis executor caspase‐3 was also gradually decreased after 48 h of gefitinib stimulation (Figure 1F,G). To further confirm whether the increase in cell number and viability on 3 days after drug administration were merely due to chemical ineffectiveness, we stimulated lung cancer cells with gefitinib twice (Figure S1A). However, we found that two doses of gefitinib did not exhibit better cytotoxic activity than one dose (Figure S1B,C). Hence, increasing the drug dosage or administering multiple doses alone cannot overcome gefitinib resistance.

### 2.2 Phenotypic Screening Strategy Identifies That Separase Inhibitor Sepin‐1 Combined With Gefitinib Markedly Promotes Lung Cancer Cell Death

**FIGURE 2:**
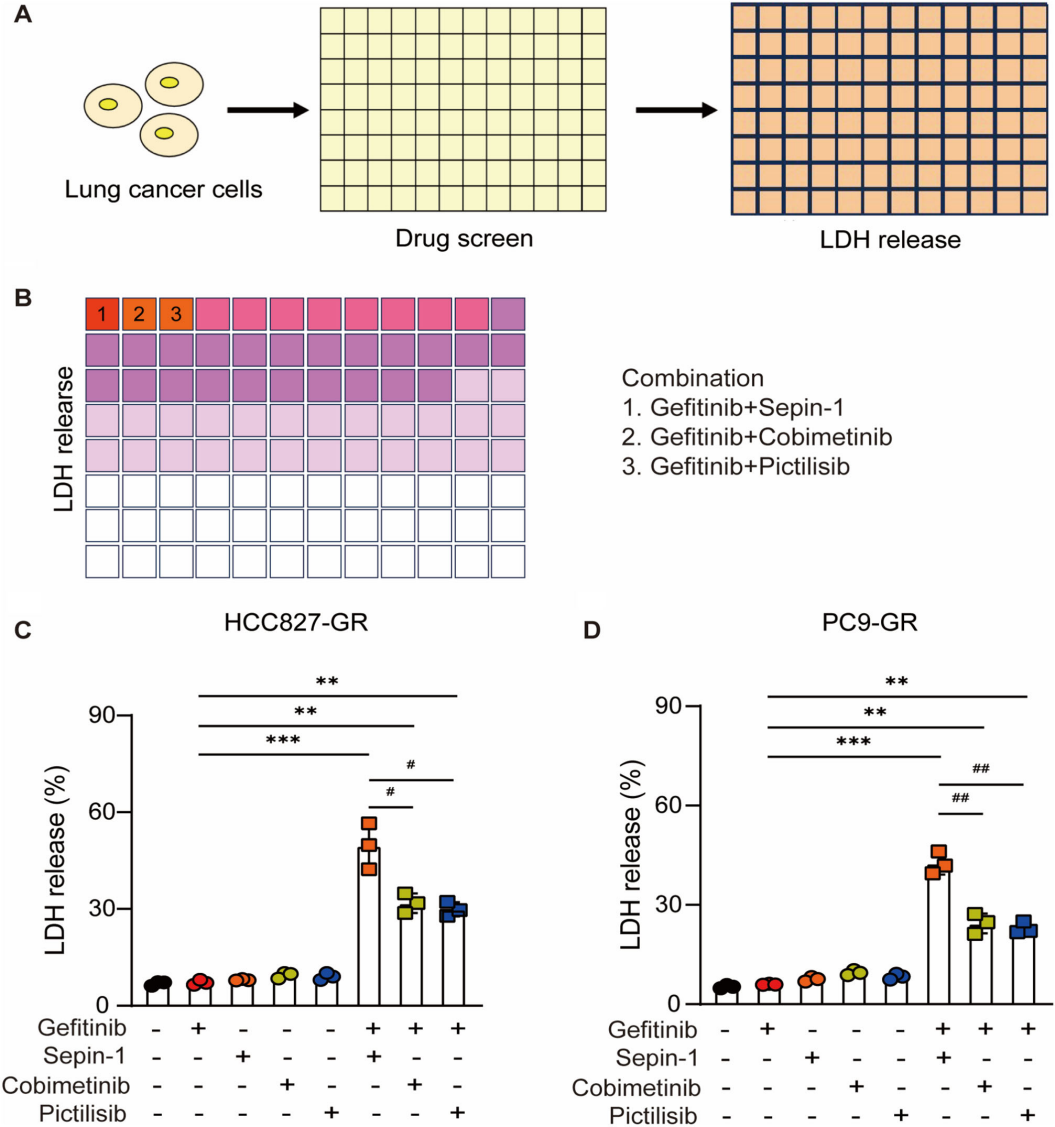
Identification of drugs that inhibit the growth of human lung cancer cells. (A) Drug screening strategy: human lung cancer cells were exposed to 60 different drug combinations for 72 h, and the amount of LDH released from human lung cancer cells was detected. (B) Heatmap of the change in HCC827‐GR and PC9‐GR human lung cancer cell activity based on the amount of LDH released. The top three drug combinations in the heatmap were gefitinib plus Sepin‐1, gefitinib plus cobimetinib, and gefitinib plus pictilisib. (C and D) The amount of LDH release in the supernatant of HCC827‐GR and PC9‐GR human lung cancer cells was determined after 72 h of stimulation with or without gefitinib (7.5 µM), Sepin‐1 (10 µM), cobimetinib (10 µM), and pictilisib (10 µM) (*n* = 3). (C and D) Graphs show the mean ± SD of replicates, representing at least three independent experiments (*p < 0.05, **p < 0.01, ***p < 0.001, ^#^ *p* < 0.05, ^##^ *p* < 0.01, two‐tailed *t*‐test).

Combination therapy is currently the primary approach to overcome single anti‐cancer drug resistance in clinical patients [24]. In lung cancer patients with gefitinib resistance, the predominant strategy against gefitinib resistance is combined with chemotherapeutics agent. However, this strategy failed to significantly extend the survival time of patients [25]. To solve this problem, we found out that the chemicals that can effectively be combined with gefitinib to enhance anti‐tumor effects are crucial. We established a phenotypic screening strategy to identify compounds that is sufficient to overcome gefitinib resistance when combined with a low dose of gefitinib (Figure 2A). By screening 60 compounds, we identified three compounds, separase inhibitor Sepin‐1, MEK1 inhibitor cobimetinib, and PI3K inhibitor pictilisib, that obviously enhanced LDH release, a marker indicating cell death, in HCC827‐GR and PC9‐GR cells (Figure 2B). While MEK1 signaling and PI3K signaling have been extensively studied in lung cancer, the role of separase in lung cancer is still unknown. Notably, the combination of separase inhibitor Sepin‐1 and gefitinib resulted in more marked LDH release than the combination of gefitinib with cobimetinib or pictilisib (Figure 2C,D). Taken together, our phenotypic screening strategy identifies separase inhibitor Sepin‐1 as a promising candidate for overcoming gefitinib resistance.

### 2.3 Inhibiting Separase Plus Gefitinib Promotes the Development of PANoptosis in Lung Cancer Cells

**FIGURE 3:**
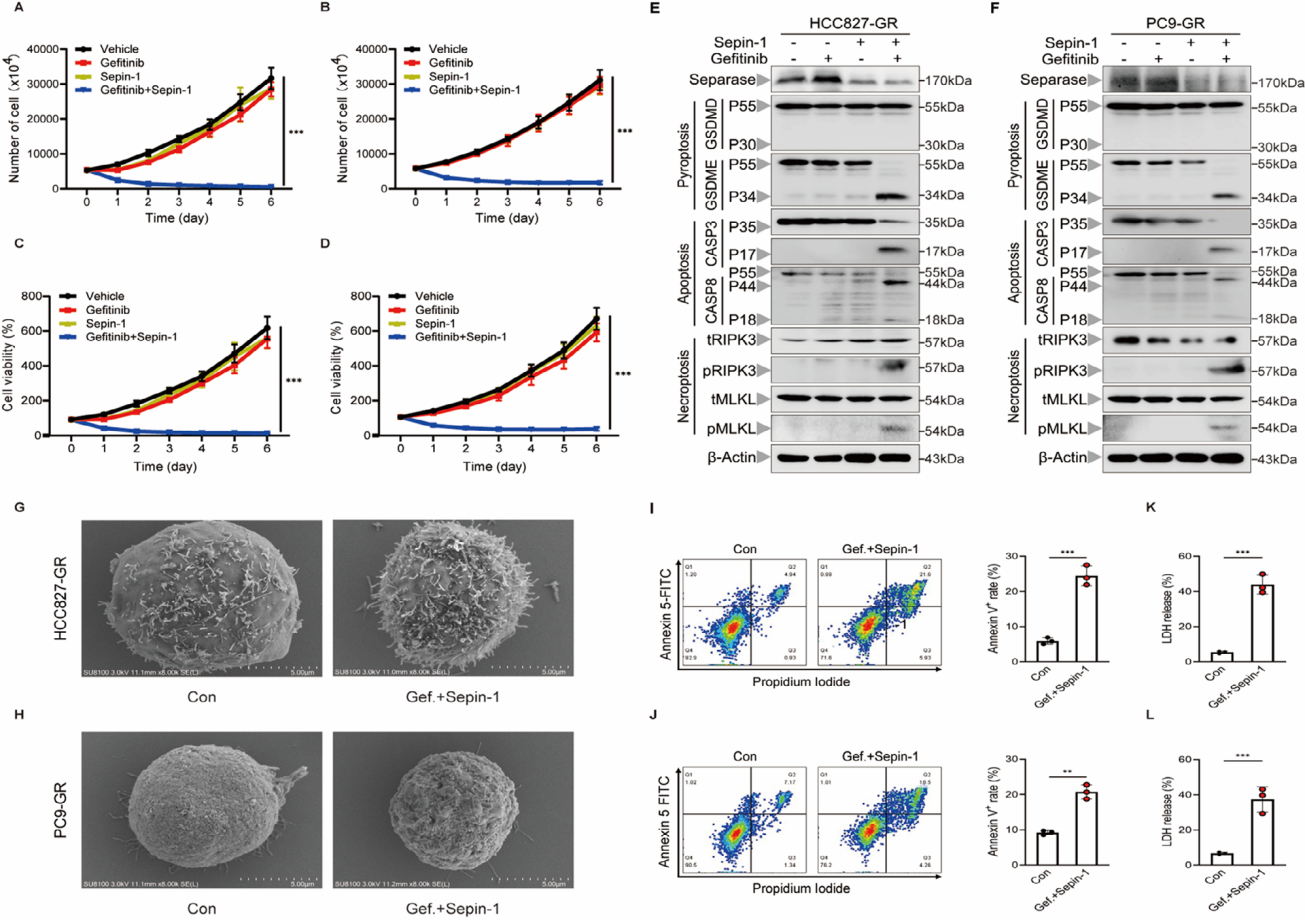
Inhibiting separase plus gefitinib promotes RIPK1‐mediated PANoptosis in lung cancer cells. (A and B) The results of the number of the HCC827‐GR (A) and PC9‐GR (B) cell lines treated with or without gefitinib (7.5 µM) and Sepin‐1 (10 µM) for 7 days (*n* = 3). (C and D) The cell viability of HCC827‐GR (C) and PC9‐GR (D) cell lines stimulated with or without gefitinib (7.5 µM) and Sepin‐1 (10 µM) for 7 days was measured using CCK‐8 assays (*n* = 3). (E and F) Immunoblot analysis of separase, pro‐ (P53) and activated (P30) GSDMD, pro‐ (P53) and activated (P34) GSDME, pro‐ (P35) and cleaved (P17) caspase‐3 (CASP3), pro‐ (P55) and cleaved (P18) caspase‐8 (CASP8), total RIPK3 (tRIPK3) and phosphorylated RIPK3 (pRIPK3), and total MLKL (tMLKL) and phosphorylated MLKL (pMLKL) in HCC827‐GR (E) and PC9‐GR (F) cell lines after stimulated with or without gefitinib (7.5 µM) and Sepin‐1 (10 µM). β‐Actin was used as the internal control. (G and H) Representative scanning electronic micrographs of HCC827‐GR (G) and PC9‐GR (H) cell lines with the treatment of gefitinib plus Sepin‐1 (*n* = 3). (I and J) Representative flow cytometry plots and quantification of HCC827‐GR (I) and PC9‐GR (J) cell lines stained with annexin‐V and propidium iodide (PI) with the treatment of gefitinib plus Sepin‐1 (*n* = 3). (K and L) LDH assay in the supernatants of HCC827‐GR (K) and PC9‐GR (L) cell lines stimulated with the treatment of gefitinib plus Sepin‐1 (*n* = 3). (A, B, I, J, K, and L) Data are shown as mean ± SD (*p < 0.05, **p < 0.01, ***p < 0.001, ****p < 0.0001, two‐tailed *t*‐test or two‐way ANOVA test). (E–H) Data are representative of at least three independent experiments.

To confirm whether separase inhibition combined with gefitinib induces long‐term growth inhibition and cytotoxic in lung cancer cells, the HCC827‐GR cells and PC9‐GR cells were treated with separase inhibitor Sepin‐1 plus low‐dose gefitinib for 7 days. Consistent with previous results, single low‐dose gefitinib or separase inhibitor Sepin‐1 failed to block the growth of lung cancer cells. But the combination presented a sustained cytotoxic effect on lung cancer cells (Figure 3A–D). To further explore how separase inhibitor Sepin‐1 combined with low‐dose gefitinib overcomes apoptosis resistance and continuously promotes cell death, we performed flow cytometry and found that this combination can effectively induce apoptosis in gefitinib‐resistant lung cancer cells (Figure 3I,J). Besides, western blotting revealed that separase inhibitor Sepin‐1 combined low‐dose gefitinib not only induces the cleavage of caspase‐3 to trigger the development of apoptosis but also simultaneously triggers the cleavage of GSDME and the phosphorylation of RIPK3 and MLKL, indicating the development of pyroptosis and necroptosis (Figure 3E,F). Furthermore, compared to untreated cells, scanning electron microscopy showed that the combination treatment led to increased pore formation and rupture of the cell membrane (Figure 3G,H). And the combination of two chemicals also induced significant LDH release in lung cancer lungs (Figure 3K,L). These results suggested that separase inhibition combined with gefitinib induces PANoptosis to effectively overcome apoptosis resistance and sustainedly kill lung cancer cells.

### 2.4 Inhibiting Separase Plus Gefitinib Promotes RIPK1‐Mediated PANoptosis in Lung Cancer Cells

**FIGURE 4:**
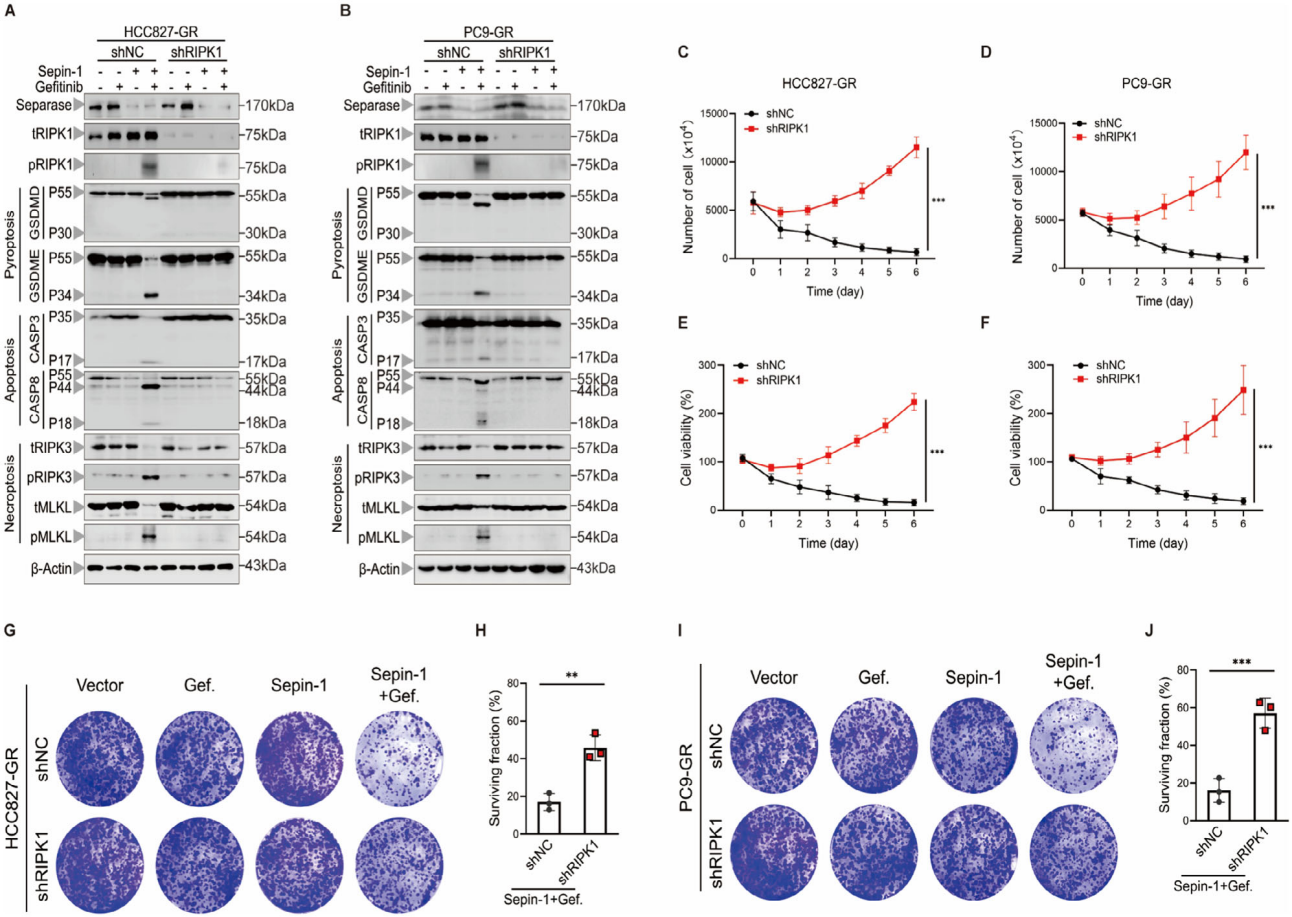
Inhibiting separase plus gefitinib promotes RIPK1‐mediated PANoptosis in lung cancer cells. (A and B) Immunoblot analysis of separase, total RIPK1 (tRIPK1), phosphorylated RIPK1 (pRIPK1), pro‐ (P55) and activated (P30) GSDMD, pro‐ (P55) and activated (P34) GSDME, pro‐ (P35) and cleaved (P17) caspase‐3 (CASP3), pro‐ (P55) and cleaved (P18) caspase‐8 (CASP8), total RIPK3 (tRIPK3) and phosphorylated RIPK3 (pRIPK3), total MLKL (tMLKL) and phosphorylated MLKL (pMLKL) in HCC827‐GR‐shNC, HCC827‐GR‐shRIPK1 (A), PC9‐GR‐shNC and PC9‐GR‐shRIPK1 (B) cell lines after treatment with or without gefitinib (7.5 µM) and Sepin‐1 (10 µM). β‐Actin was used as the internal control. (C and D) The results of the number of the HCC827‐GR‐shNC, HCC827‐GR‐shRIPK1 (C), PC9‐GR‐shNC, and PC9‐GR‐shRIPK1 (D) cell lines treated with gefitinib (7.5 µM) and Sepin‐1 (10 µM) for 7 days (*n* = 3). (E and F) The cell viability of HCC827‐GR‐shNC and HCC827‐GR‐shRIPK1 (E), and PC9‐GR‐shNC and PC9‐GR‐shRIPK1 (F) cell lines stimulated with gefitinib (7.5 µM) and Sepin‐1 (10 µM) for 7 days was measured using CCK‐8 assays (*n* = 3). (G–J) Representative results of the colony formation showed the cell proliferation in the HCC827‐GR‐shNC and HCC827‐GR‐shRIPK1 (G and H), and PC9‐GR‐shNC and PC9‐GR‐shRIPK1 (I and J) cell lines treated with or without gefitinib (7.5 µM) and Sepin‐1 (10 µM) for 2 weeks (*n* = 3). (C–J) Data are shown as mean ± SD (*p < 0.05, **p < 0.01, ***p < 0.001, two‐tailed *t*‐test or two‐way ANOVA test). (A and B) Data are representative of at least three independent experiments.

RIPK1 is a crucial mediator in the development of PANoptosis. To further verify that the combination of separase inhibition and gefitinib induces PANoptosis via RIPK1, RIPK1 shRNA was transfected into HCC827‐GR cells and PC9‐GR cells to successfully silence RIPK1 expression. Subsequently, we stimulated RIPK1‐silenced lung cancer cells with separase inhibitor Sepin‐1 and gefitinib. Results showed that knockdown of RIPK1 suppressed the development of PANoptosis by inhibiting the activation of caspase‐3, GSDME, RIPK3, and MLKL (Figure 4A,B). Moreover, to confirm whether inhibiting RIPK1‐mediated PANoptosis impacts the efficacy of combination therapy, we assessed the growth ability of RIPK1‐silenced lung cancer cells after Sepin‐1 and gefitinib stimulation. As expected, knockdown of RIPK1 effectively reduced combination therapy‐mediated cell death in lung cancer cells (Figure 4C–F). Similar results were also corroborated by clonogenic survival assays, which showed that the clonogenic potential of HCC827‐GR cells and PC9‐GR cells with RIPK1 silencing was restored after exposure to Sepin‐1 and gefitinib (Figure 4G–J). Taken together, these results suggested that the combination therapy of Sepin‐1 and gefitinib can sustainably kill lung cancer cells through RIPK1‐mediated PANoptosis.

### 2.5 Loss of TAK1 Mediates Separase Deficiency Plus Gefitinib‐Induced PANoptosis

**FIGURE 5:**
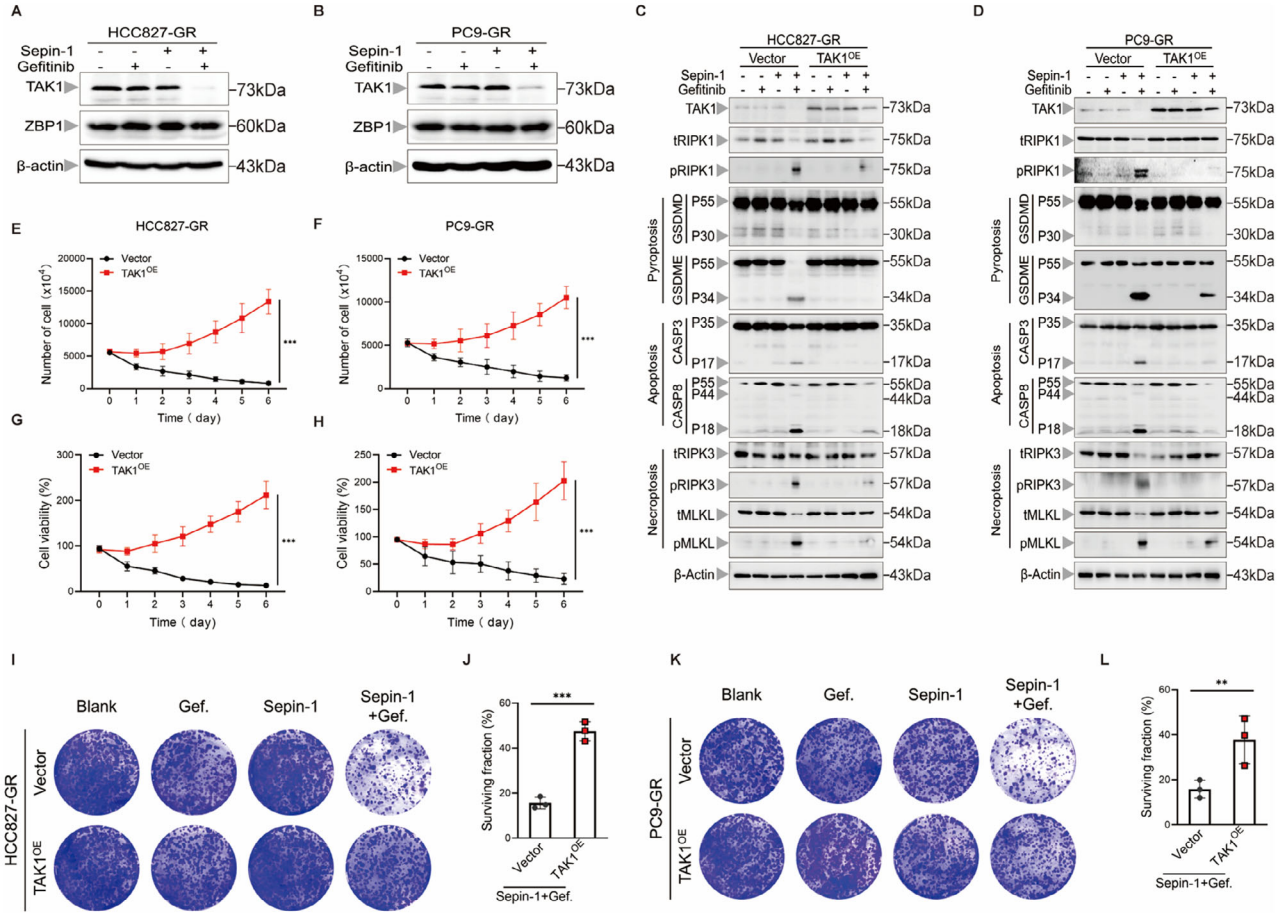
TAK1 mediates separase deficiency plus gefitinib‐induced PANoptosis. (A and B) Immunoblot analysis of TAK1 and ZBP‐1 in HCC827‐GR(A) and PC9‐GR(B) after treatment with or without gefitinib (7.5 µM) and Sepin‐1 (10 µM). β‐Actin was used as the internal control. (C and D) Immunoblot analysis of TAK1, total RIPK1 (tRIPK1), phosphorylated RIPK1 (pRIPK1), pro‐ (P55) and activated (P30) GSDMD, pro‐ (P55) and activated (P34) GSDME, pro‐ (P35) and cleaved (P17) caspase‐3 (CASP3), pro‐(P55) and cleaved (P18) caspase‐8 (CASP8), total RIPK3 (tRIPK3) and phosphorylated RIPK3 (pRIPK3), total MLKL (tMLKL), and phosphorylated MLKL (pMLKL) in HCC827‐GR and HCC827‐GR‐TAK1^OE^ (C), and PC9‐GR and PC9‐GR‐TAK1^OE^ (D) cell lines after treatment with or without gefitinib (7.5 µM) and Sepin‐1 (10 µM). β‐Actin was used as the internal control. (E and F) The results of the number of the HCC827‐GR and HCC827‐GR‐TAK1^OE^ (E), and PC9‐GR and PC9‐GR‐ TAK1^OE^ (F) cell lines treated with gefitinib (7.5 µM) and Sepin‐1 (10 µM) for 7 days (*n* = 3). (G and H) The cell viability of HCC827‐GR and HCC827‐GR‐TAK1 ^OE^ (G) and PC9‐GR and PC9‐GR‐ TAK1 ^OE^ (H) cell lines stimulated with gefitinib (7.5 µM) and Sepin‐1 (10 µM) for 7 days (*n* = 3). (I–L) Representative results and quantification of the colony formation showed the cell proliferation in the HCC827‐GR‐VECTOR and HCC827‐GR‐TAK1^OE^ (I and J), and PC9‐GR‐VECTOR and PC9‐GR‐ TAK1^OE^ (K, L) cell line treated with or without gefitinib (7.5 µM) and Sepin‐1 (10 µM) for 2 weeks (*n* = 3). (E–L) Data are shown as mean ± SD (*p < 0.05, **p < 0.01, ***p < 0.001, ****p < 0.0001, two‐tailed *t*‐test or two‐way ANOVA test). (A–D) Data are representative of at least three independent experiments.

Previous studies have identified that ZBP1 and TAK1 are important sense proteins in regulating RIPK1 activation [26, 27]. To explore the role of ZBP1 and TAK1 in combined stimulation, we first examined the expression of ZBP1 and TAK1 in PC9‐GR and HCC827‐GR cells after Sepin‐1 and gefitinib stimulation. The results revealed that, following treatment with separase inhibitor Sepin‐1 and gefitinib, the expression of TAK1 was significantly inhibited, while the expression of ZBP1 remained unaffected (Figure 5A,B). This indicates TAK1 is more likely the key target for regulating separase deficiency plus gefitinib‐induced PANoptosis. To further demonstrate that TAK1 indeed participated in separase deficiency plus gefitinib‐mediated PANoptosis, TAK1 was overexpressed TAK1 in HCC827‐GR cells and PC9‐GR cells by lentivirus transfection. Overexpression of TAK1 suppressed the activation of RIPK1, which further inhibited the development of PANoptosis by inhibiting caspase‐3, GSDME, RIPK3, and MLKL activation (Figure 5C,D). Additionally, overexpression of TAK1 also inhibits combination stimulation‐mediated cell death in lung cancer cells (Figure 5E–H). Colony formation assay further certified this result. The clonogenic potential of TAK1‐overexpressing HCC827‐GR and PC9‐GR was minimally affected by exposure to Sepin‐1 and gefitinib treatment (Figure 5I–L). To further confirm that TAK1 mediates gefitinib plus Sepin‐1‐induced cell through RIPK1, we stimulated RIPK1‐silenced lung cancer cells with TAK1 inhibitor 5z7. We found that inhibited TAK1 did not sufficiently inhibit cell growth in RIPK1‐silenced lung cancer cells (Figure S2A,B). These results suggested that TAK1 is an indispensable upstream mediator to participate in separase inhibition plus gefitinib‐mediated PANoptosis.

### 2.6 PTBP1 is Involved in Separase Deficiency Plus Gefitinib‐Induced TAK1 Deficiency

The ubiquitin‐proteasome degradation is an important pathway in inducing TAK1 deficiency. Previous studies have reported that different pathway proteins, including TNIP3, FGFR, and PTBP1, can induce TAK1 deficiency by regulating the ubiquitin‐proteasome degradation pathway [28, 29, 30]. Hence, to identify which of these proteins is involved in combined stimulation‐induced TAK1 deficiency, we detected their RNA expression by real‐time PCR. Our results revealed that only the mRNA expression level of PTBP1 was significantly reduced (Figure S3A). To further explore the role of PTBP1, we further overexpressed PTBP1 in lung cancer cells and then subjected them to combined stimulation. We found that overexpression of PTBP1 significantly inhibits combined stimulation‐induced deficiency of TAK1 expression and decrease in cell viability (Figure S3B–E). Besides, western blot analysis revealed that the biomarkers of PANoptosis were inactivated with PTBP1 overexpression (Figure S3D,E). These results suggest that PTBP1 plays a vital role in mediating separase deficiency plus gefitinib‐induced TAK1 deficiency and PANoptosis development.

### 2.7 Suppressing Separase Expression Enhances Anti‐Tumor Effect of Gefitinib Treatment in BALB/c Nude Mice With Lung Cancer Xenograft Models

**FIGURE 6:**
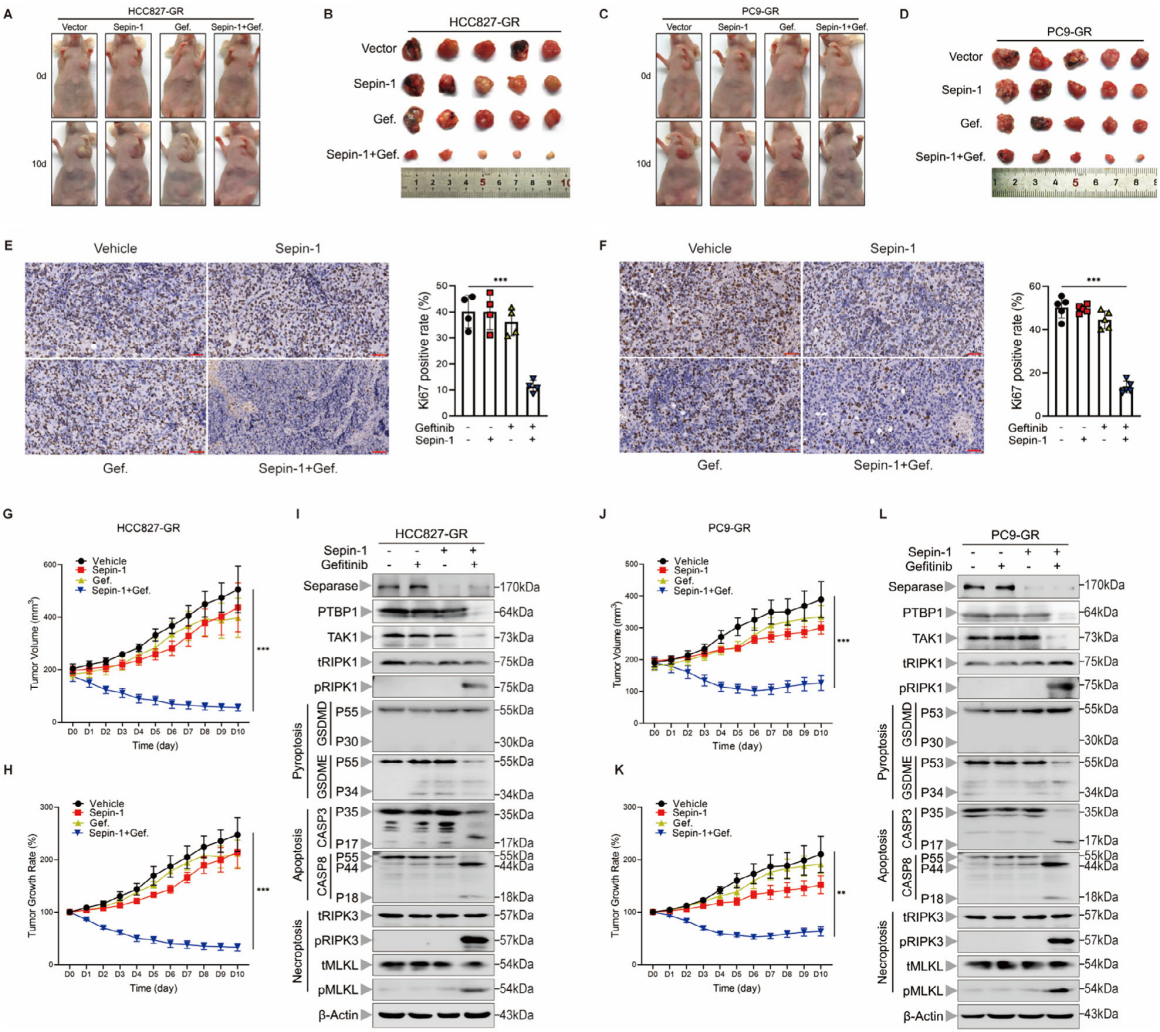
Suppressing separase expression enhances anti‐tumor effect of gefitinib treatment in BALB/c nude mice with lung cancer xenograft models. (A and C) Representative imaging of nude mice was injected subcutaneously with the HCC827‐GR (HCC827‐GR model) and PC9‐GR cell lines (PC9‐GR model), followed by treatment with or without gefitinib (25 mg/kg) and Sepin‐1 (10 mg/kg), and tumor size was observed in nude mice for 10 consecutive days. (B and D) Photographs of the excised tumors from HCC827‐GR (B) and PC9‐GR (D) models, followed by treatment with or without gefitinib (25 mg/kg) and Sepin‐1(10 mg/kg) for 10 days (HCC827‐GR, *n* = 5; PC9‐GR, *n* = 5) and comparison of the tumor sizes. (E and F) Representative IHC staining images of Ki67. Scale bar = 50 µm, and IHC staining scores of Ki67 expression in HCC827‐GR model (E) and PC9‐GR model (F), followed by treatment with or without gefitinib (25 mg/kg) and Sepin‐1 (10 mg/kg) for 10 days. (G and J) Statistical analysis of the tumor volumes in nude mice from the HCC827‐GR (G) and PC9‐GR (J) models, followed by intraperitoneal injection with or without gefitinib (25 mg/kg) and Sepin‐1 (10 mg/kg) for 10 days. (H and K) Statistical analysis of the tumor growth rate in nude mice from the HCC827‐GR (H) and PC9‐GR (K) models, followed by treatment with or without gefitinib (25 mg/kg) and Sepin‐1(10 mg/kg) for 10 days. (I and L) Immunoblot analysis of separase, PTBP1, TAK1, total RIPK1 (tRIPK1), phosphorylated RIPK1 (pRIPK1), pro‐ (P55) and activated (P30) GSDMD, pro‐ (P55) and activated (P34) GSDME, pro‐ (P35) and cleaved (P17) caspase‐3 (CASP3), pro‐(P55) and cleaved (P18) caspase‐8 (CASP8), total RIPK3 (tRIPK3) and phosphorylated RIPK3 (pRIPK3), and total MLKL (tMLKL) and phosphorylated MLKL (pMLKL) in HCC827‐GR (I) and PC9‐GR (L) model after treatment with or without gefitinib (25 mg/kg) and Sepin‐1 (10 mg/kg). β‐Actin was used as the internal control. (A–K) Data are shown as mean ± SD (*p < 0.05, **p < 0.01, ***p < 0.001, two‐tailed *t*‐test; *n*  =  4 or 5). (I and L) Data are representative of at least three independent experiments.

Subsequently, we injected HCC827‐GR and PC9‐GR cells into BALB/c nude mice to establish lung cancer xenograft model. After the models were established, the mice were administered Sepin‐1 and gefitinib, either separately or in combination, once a day for 10 days. Consistent with our in vitro results, treatment with gefitinib or Sepin‐1 alone is insufficient to hinder the growth of lung cancer xenograft tumor. However, the combination of separase inhibitor Sepin‐1 plus gefitinib prominently reduced xenograft tumor volume (Figure 6A–D). We also evaluated the expression of Ki67, a marker of abnormal cell proliferation, in the xenograft tumor tissues. The results suggested that the combination therapy can significantly inhibit the expression of Ki67 in lung cancer tissues (Figure 6E,F). Remarkably, the combination therapy continuously exhibits a potent cytotoxic effect against lung cancer tumors after multiple administrations (Figure 6G,H,J,K). Moreover, we further investigate whether Sepin‐1 combined with gefitinib also promotes the regression of lung cancer tumors in vivo by inducing the development of PANoptosis. We performed western blotting and found that combination therapy induces the development of PANoptosis by promoting the activation of caspase‐8, caspase‐3, GSDME, RIPK3, and MLKL in lung cancer xenograft. Additionally, Sepin‐1 plus gefitinib administration also inhibited the expression of PTBP1 and TAK1, and the activation of RIPK1 in vivo (Figure 6I,L). These results suggested that the combination therapy of Sepin‐1 plus gefitinib is sufficient to promote the regression of lung cancer xenograft tumor by triggering PANoptosis via PTBP1/TAK1/RIPK1 pathway.

### 2.8 Separase Expression Is Linked With the Survival Time of Lung Cancer in Patients

**FIGURE 7:**
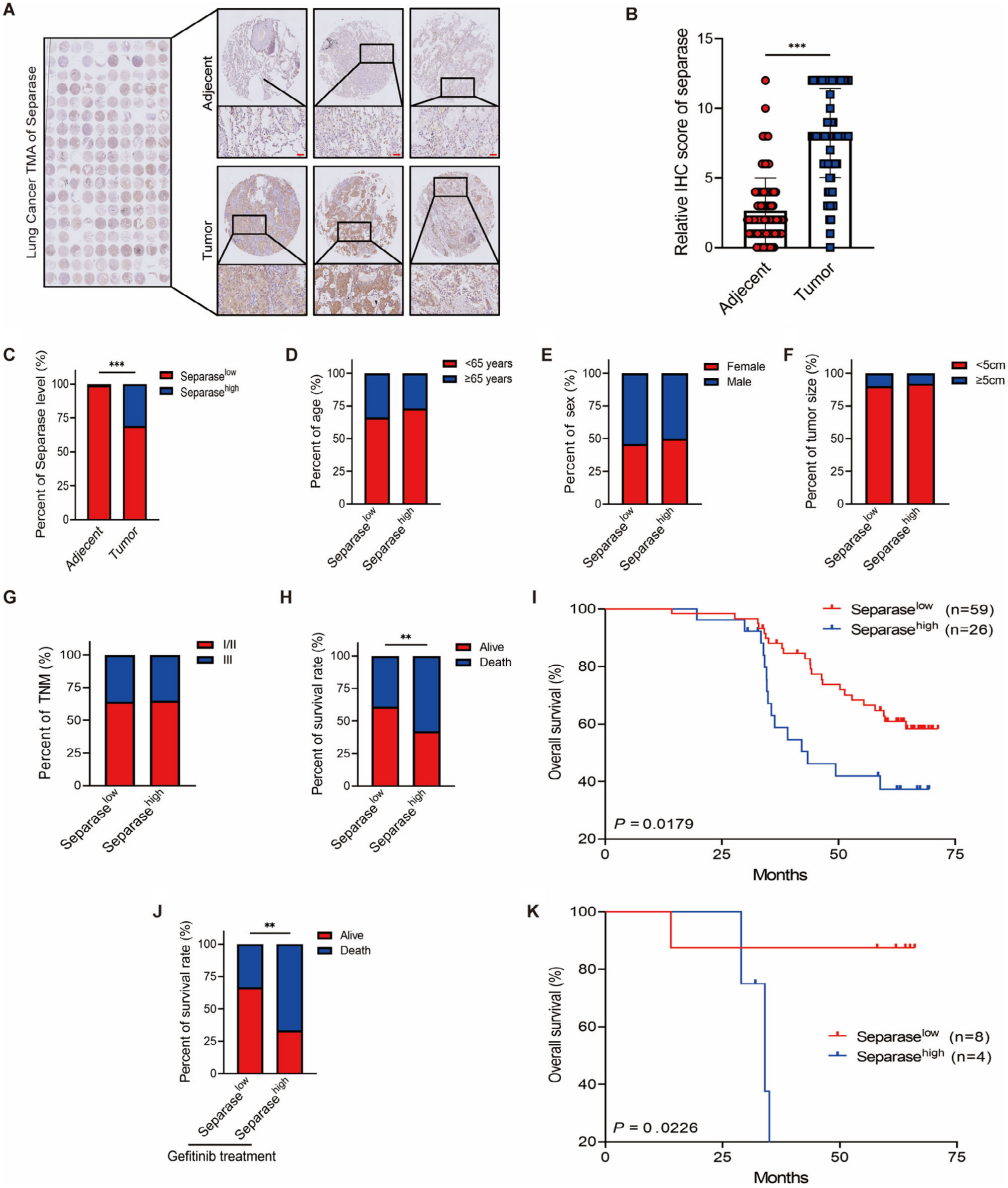
Separase expression is linked with the survival time of lung cancer in patients. (A) The human lung cancer tissues and adjacent normal tissues in TMA were stained immunohistochemically for separase (*n* = 85). (B) The relative IHC staining scores of separase expression in lung cancer tissues and adjacent normal tissues from TMA (*n* = 85). (C) The different expression level of separase in human lung cancer tissues and adjacent normal tissues (*n* = 85). (D–H) Separase expression correlated with age (D), sex (E), tumor size (F), TNM (G), and survival rate (H) in lung cancer patients (*n* = 85). (I) The correlation between separase expression (Separase^low^, *n* = 59; Separase^high^, *n* = 26) and the survival time of lung cancer patients was presented by Kaplan–Meier survival analysis. (J) Separase expression correlated with survival rate in lung cancer patients with gefitinib treatment (*n* = 12). (K) The correlation between separase expression (Separase^low^, *n* = 8; Separase^high^, *n* = 4) and the survival time of lung cancer patients with gefitinib treatment was presented by Kaplan–Meier survival analysis. (B–H and J) data are shown as mean ± SD (*p < 0.05, **p < 0.01, ***p < 0.001, two‐tailed *t*‐test or two‐way ANOVA test).

Although our results suggest that higher expression of separase impedes the efficacy of lung cancer treatment, it remains unclear whether high expression of separase is associated with a worse prognosis in clinical lung cancer patients. To address this, we performed immunohistochemical analysis of separase in lung cancer tissue microarray (TMA) and quantified separase expression in paired normal lung and lung carcinoma. Compared to adjacent tissues, the expression of separase in adjacent tissues of the same patient is much lower than that in tumor tissues (Figure 7A,B). Next, according to the staining score of separase in the tumor tissues, the lung cancer patients were further divided into two groups. In contrast to paracancerous tissues that hardly expressed separase, the expression of separase was specifically elevated in cancerous tissues from 31% lung cancer patients (Figure 7C). However, increased separase levels were not linked with bigger tumor volume, more advanced clinical stages of NSCLC, and the age or sex of NSCLC patients (Figure 7D–G). Nevertheless, Kaplan–Meier survival analyses revealed increased risk of lung cancer‐related death in patients with high expression of separase (Figure 7H,I). To further confirm the role of separase in lung cancer patients with gefitinib treatment, we selected the patients with gefitinib treatment in above samples and found that high expression of separase is linked with higher death rate and shorter survival time in lung cancer patients with gefitinib treatment (Figure 7J,K). These results suggested that higher expression of separase is associated with shorter survival time in clinical lung cancer patients. Moreover, higher separase expression is linked with worse gefitinib efficacy in lung cancer patients.

### 2.9 p‐PIRK3 Is Associated With the Progress of Lung Cancer Patients

**FIGURE 8:**
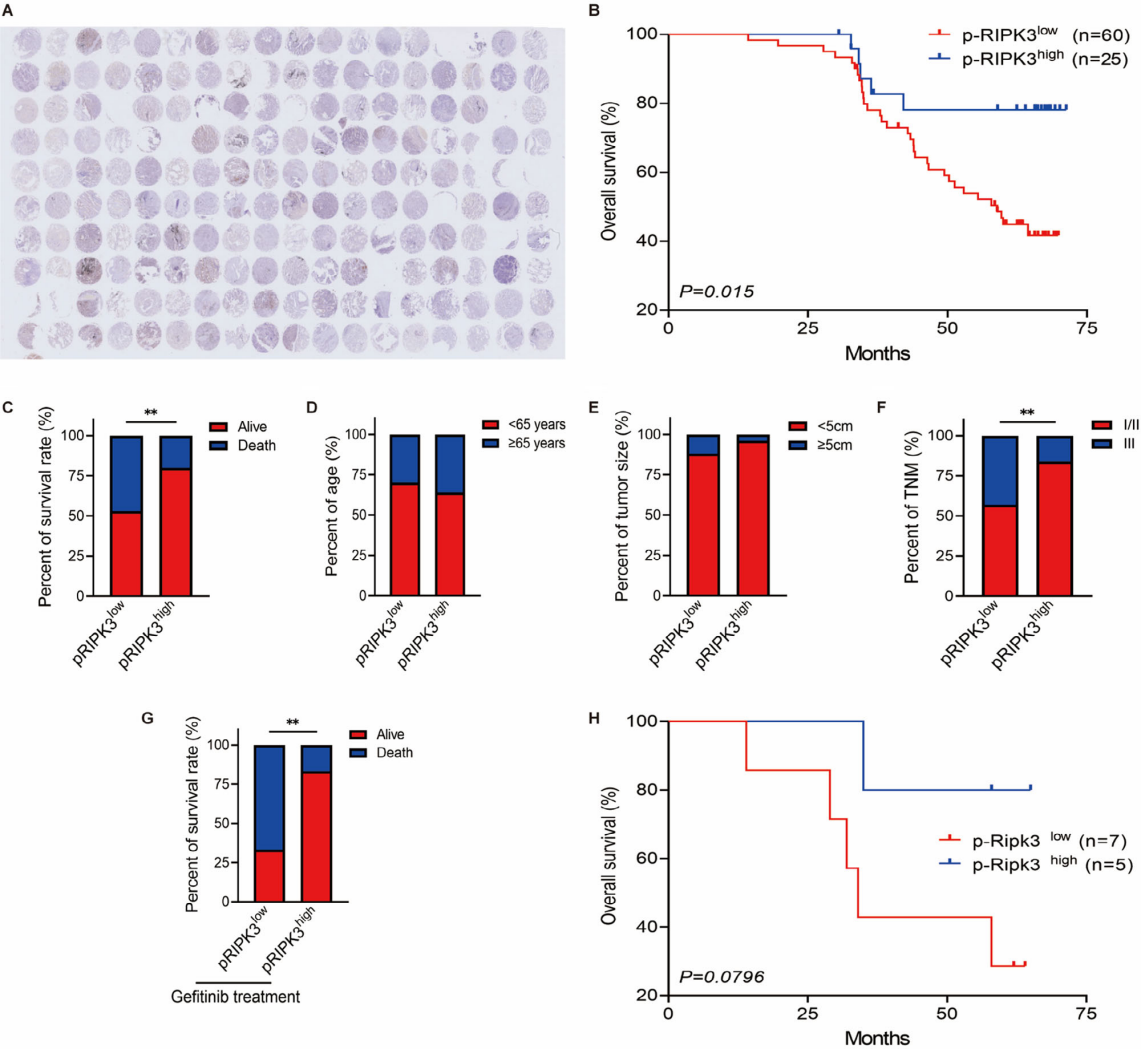
p‐PIRK3 is associated with the progress of lung cancer patients. (A) The human lung cancer tissues and adjacent normal tissues in TMA were stained immunohistochemically for p‐RIPK3 (*n* = 85). (B) The correlation between p‐RIPK3 expression (p‐RIPK3^low^, *n* = 60; p‐RIPK3^high^, *n* = 25) and the survival time of lung cancer patients was presented by Kaplan–Meier survival analysis. (C–F) p‐RIPK3 expression correlated with the survival rate (C), age (D), tumor size (E), and TNM (F) in lung cancer patients (*n* = 85). (G) p‐RIPK3 expression correlated with survival rate in lung cancer patients with gefitinib treatment (*n* = 12). (H) The correlation between p‐RIPK3 expression (p‐RIPK3^low^, *n* = 7; p‐RIPK3^high^, *n* = 5) and the survival time of lung cancer patients with gefitinib treatment was presented by Kaplan–Meier survival analysis. (C–G) Data are shown as mean ± SD (*p < 0.05, **p < 0.01, ***p < 0.001, two‐tailed *t*‐test or two‐way ANOVA test).

To further confirm whether the development of PANoptosis is linked with the disease process of lung cancer patients, the activity of RIPK3, as one of the PANoptosis markers, was also measured by immunohistochemistry in lung cancer tissues from the same lung cancer patients (Figure 8A). Results showed that lung cancer patients with high p‐RIPK3 expression had significantly longer survival times than those with low p‐RIPK3 expression (Figure 8B). At the end point, compared with only 20% of patients with low p‐RIPK3 expression, 47% of patients with high p‐RIPK3 expression survived (Figure 8C). In addition, the expression level of p‐RIPK3 was also independent of the age of lung cancer patients, but p‐RIPK3 expression was lower in larger and more malignant lung cancer tumors (Figure 8D–F). We also found that lower p‐RIPK3 is linked with higher death rate and shorter survival time in lung cancer patients with gefitinib treatment (Figure 8G,H). Taken together, our results suggested that p‐RIPK3 is correlated with the progress of lung cancer patients. Loss of p‐RIPK3 is also linked with the poorer therapeutic effect of gefitinib in lung cancer patients.

## 3 Discussion

This work establishes the critical role of separase deficiency in promoting gefitinib‐triggered lung cancer cell death via PANoptosis. Gefitinib, the earliest used EGFR‐TKi, is the first‐line drug for advanced lung cancer patients with EGFR mutations. Compared to chemotherapy drugs, lung cancer patients treated with gefitinib will have a longer progression‐free survival (PFS). For lung cancer patients after surgical treatment, gefitinib also markedly extended the short‐term disease‐free survival (DFS). However, in terms of 5‐year DFS, adjuvant gefitinib therapy did not show better results. Moreover, gefitinib treatment failed to prolong the overall survival (OS), indicating that long‐term use of gefitinib will lead to drug resistance. Our results also support this evidence, as using gefitinib alone only suppress lung cancer cell growth for a short period of time.

Since high‐dosage use of gefitinib is easy to cause the occurrence of adverse effects, such as hepatic impairment and fatal liver failures [31, 32]. Currently, the primary approach in clinical treatment to overcome gefitinib resistance is combined with chemotherapy drugs. Although gefitinib plus chemotherapy results in higher disease control rates (DCR) and longer PFS compared to monotherapy of chemotherapy medicine for a part of lung cancer patients, the other part of patients with treatment failure does not exhibit superior PFS after combined treatment [4, 5, 33]. The main reason for this phenomenon maybe due to gefitinib plus chemotherapy that still induces apoptosis in lung cancer cells, which is also susceptible to the development of apoptosis resistance [6]. Recent evidence indicates that chemotherapy can provoke pyroptosis in cancer cells with high GSDME‐expression, but additional clinical evidence is required to support this role [34, 35]. Here, we discovered that suppressing separase expression can significantly enhance the sensitivity of lung cancer cells to gefitinib by triggering apoptosis, necroptosis, and pyroptosis, namely, as PANoptosis.

PANoptosis was first identified in cells infected with influenza A virus (IAV) [36]. IAV induces Z‐DNA‐binding protein 1 (ZBP1) to form the ZBP1‐panoptosome complex; activates the pyroptotic‐related protein caspase‐1 and GSDMD, apoptotic markers caspase‐8, caspase‐3, and caspase‐7, and necroptotic labels RIPK1, RIPK3, and MLKL; and induces PANoptosis [37]. Similarly, absent in melanoma 2 (AIM2) as a cytosolic dsDNA sensor can also be found to mediate the formation of AIM2‐PANoptosome complex [26]. In response to *Francisella novicida* and herpes simplex virus 1 (HSV1) infections, AIM2 recruits and activates pyrin, ASC, caspase‐1, caspase‐8, FADD, RIPK1, and RIPK3 to induce PANoptosis [38]. In addition, TAK1 inhibition by Yersinia infection, genetic mutation, or TAK1 inhibitors also can induce PANoptosis [16]. Previous studies demonstrated that ZBP1 and AIM2 are capable of triggering PANoptosis in cancer cells [14, 39], but our results revealed that TAK1 inhibition is the primary mechanism to mediate separase inhibition plus gefitinib treatment‐induced PANoptosis. In this process, RIPK1, RIPK3, MLKL, caspase‐3, caspase‐8, and GSDME are assembled to form PANoptosome.

In immune cells, merely inhibiting one type of programmed cell death is difficult to completely suppress cell death, which contributes to the activation of alternative cell death pathways. Co‐knock out of multiple cell death executor is more efficient to suppress infection‐mediated macrophage death [26]. Currently available chemotherapy mainly promotes cell apoptosis in carcinoma cells [40]. However, it is a loss of the feedback‐pathway to trigger other cell death in cancer cells when apoptosis resistance occurs. Therefore, the manner to simultaneously induce the development of apoptosis and other types of programmed cell death is crucial to eliminate cancer cells. Our results suggest that separase deficiency plus gefitinib treatment is a superior anti‐cancer strategy to sustained killing of cancer cells by inducing the activation of apoptotic executor caspase‐3, pyroptotic executor GSDME, and necroptotic executor MLKL, which trigger the occurrence of PANoptosis.

Separase controls the separation of sister chromatids to influence the process of mitosis, which is playing an indispensable role in regulating cell growth [17]. It has been observed that separase is highly expressed in several kinds of cancer cells, such as breast cancer, uterine cancer, and lung cancer [19, 20, 21]. The elevated expression of separase may be a significant factor contributing to the rapid growth of cancer cells. Inhibiting the expression of separase suppresses the growth of breast cancer cells [19, 41]. In our findings, inhibiting separase expression also delays the growth of lung cancer cells but fails to destroy lung cancer cell‐derived xenograft by inducing cell death. However, while combined with gefitinib, separase inhibition promotes cell death in lung cancer cells and leads to the regression of lung cancer tissues. This suggests that inhibiting separase may be a promising approach to enhance the effectiveness of targeted therapy for lung cancer. Nevertheless, separase inhibition is only combined with gefitinib. The role of separase deficiency with other EGFR‐TKIs in anti‐lung cancer cell growth is lack of understanding. Taken together, this study provides a novel strategy to improve the efficacy of gefitinib by facilitating lung cancer cell PANoptosis.

## 4 Methods

### 4.1 TMA Analysis

The customize clinical lung cancer TMA is commercially bought from Shanghai Outdo Biotech Company. All the patients’ clinical characteristics were assessed by Shanghai Outdo Biotech Company. All specimens were handled anonymously following the company's protocol and ethical standards. Fresh‐frozen tumor samples were obtained from patients during surgery, and formalin‐fixed and paraffin‐embedded (FFPE) sections were collected during pathological examination. Paraffin‐embedded sections were immunolabeled with separase (Abcam, ab16170) and pRIPK3 (Abcam, ab16170) antibody. Details of patient information are shown in Table S1.

### 4.2 Cell Culture and Reagents

HCC827‐GR and PC9‐GR cell lines were obtained from Cellbank of Chinese Academy of Sciences. Cells were cultured in DMEM (Gibco) medium, supplemented with 10% fetal bovine serum (Gibco), 100 U/mL penicillin, and 0.1 mg/mL streptomycin at 37°C and 5% CO_2_. Gefitinib (Selleck, S1025), cobimetinib (Selleck, S8041), pictilisib (Selleck, S1065), separase inhibitor Sepin‐1 (MedChemExpress, HY‐117522), and TAK1 inhibitor 5z7 (Sigma‐Aldrich, O9890) were reconstituted in the indicated solution following the manufacturer's instructions and used to incubate cells at the indicated concentration.

### 4.3 Chemicals and Compound Library

We utilized the customized Selleck library (Selleck), a compilation of compounds that have diverse functions, structures, and cellular targets (Table S2). The chemicals in the library were at a concentration of 10 mM (stock) in DMSO. All compounds were used at 10 µM with 7.5 µM gefitinib. The amount of LDH release was detected after combined stimulation for 48 h.

### 4.4 Lentivirus Transfection

To establish stable overexpressed TAK1 or knockdown of RIPK1 expression cells, cells (HCC827‐GR and PC9‐GR) were seeded onto six‐well plates at a density of 2 × 10^5^ cells/well and transfected with lentivirus (MOI = 20) containing TAK1 cDNA or RIPK1 shRNA (sequence: 5′‐AGGTCATGTTCTTTCAGCTTA‐3′). Successfully infected cells were used for the treatment in the following experiment.

### 4.5 Cell Count

Cell counts were conducted using an Invitrogen Countess II automated cell counter (Thermo Fisher Scientific, USA). The trypsinized cells were resuspended to 100 µL with medium. A 10 µL aliquot of this suspension was placed onto a counting slide. The cell counter was programmed to “quick count” mode, and each sample was counted three times independently to guarantee both accuracy and reproducibility.

### 4.6 Cell Viability Assay

Cell viability was quantified using the Cell Counting Kit‐8 (CCK‐8, Dojindo Laboratories, Kumamoto, Japan). Approximately 3000 HCC827‐GR cells or PC9‐GR cells were seeded per well in a 96‐well plate. The cells were incubated at 37°C and the absorbance at 450 nm was measured daily over six consecutive days to assess cell viability.

### 4.7 Colony Formation Assay

The indicated cells were seeded into six‐well plates at a density of 5 × 10^2^ cells per well. The medium was exchanged every 3 days for 2 weeks. Colonies were fixed with formalin and stained with 0.25% crystal violet, and colonies with more than 30 cells were included in the quantification. The colony number was determined by counting stained colonies using ImageJ 1.52 v (National Institutes of Health, Bethesda, MD, USA).

### 4.8 LDH Assays

LDH was performed using the CyQUANT LDH cytotoxicity assay (Thermo Fisher) according to the manufacturer's instructions.

### 4.9 qPCR Assay

qPCR for TNIP3, FGFR, and PTBP1 mRNA was performed according to the manufacturer's instructions and presented relative to BETA‐ACTIN (primer sequences, TNIP3: F‐ATTGCCGCAGAAAGTTCTACG, R‐GTCCAGTTTCGTCTTCAGCTC; FGFR: F‐CCCGTAGCTCCATATTGGACA, R‐TTTGCCATTTTTCAACCAGCG; PTBP1: F‐AGCGCGTGAAGATCCTGTTC, R‐ CAGGGGTGAGTTGCCGTAG).

### 4.10 Western Blotting

Western blotting was used to analyze protein expression as described previously. In brief, cells or tissue samples were collected and lysed in RIPA buffer containing proteinase inhibitors (Roche) and phosphatase inhibitors (Roche). The protein extracts (30 µg/lane) were separated on SDS‐PAGE gels, followed by electro‐transfer onto polyvinylidene fluoride membranes (Sigma‐Aldrich). After blocking with 5% fat‐free milk for 2 h at room temperature, the membranes were incubated with primary antibodies and subsequently incubated with secondary antibody (Santa Cruz Biotechnology). Finally, bands were scanned with Bio‐Rad Imager and individual band intensity was determined with the software ImageJ. Antibodies against the following proteins were used: separase (Abcam, ab16170), RIPK1 (CST, #3493), pRIPK1 (CST, #31122), RIPK3 (CST, #95702), pRIPK3 (Abcam, ab195117), GSDMD (Abcam, ab209845), GSDME (Abcam, ab215191), caspase‐8 (CST, #4927), cleaved caspase‐8 (CST, #8592), caspase‐3 (CST, #9662), cleaved caspase‐3 (CST, #9661), MLKL (CST, #37705), pMLKL (CST, #37333), TAK1 (Abcam, ab109526), Ptbp1 (Abcam, ab133734), and β‐actin (CST #3700).

### 4.11 Tumor Xenograft Models

Indicated HCC827‐GR and PC9‐GR cells (5 × 10^6^ cells suspended in 100 µL PBS) were subcutaneously implanted in the left underarms of 4‐week‐old BALB/c nude mice. While tumor sizes reached approximately 200–400 mm^3^, mice were randomly distributed into different groups. Mice were received as either vehicle control, gefitinib alone, Sepin‐1 alone, or gefitinib and Sepin‐1 together. Gefitinib was administered once per day by oral gavage (25 mg/kg). Sepin‐1 was administered once per day via intravenous injection (10 mg/kg). Tumor size was measured every day by vernier caliper and calculated as length × width^2^ × 0.5. All mice were sacrificed by carbon dioxide inhalation in a euthanasia chamber at the indicated time point. Tumor tissues and blood samples were collected for further examination.

### 4.12 Immunohistochemistry

Paraffin‐embedded xenograft sections were immunolabeled with Ki67 (Abcam, ab15580) antibody. Ki67 expression was calculated by multiplying the percentage of positivity by the staining score in each sample.

### 4.13 Statistical Analysis

Statistical analysis was performed with GraphPad Prism software, and data are presented as mean ± SD. In all experiments, comparisons between two groups were based on unpaired Student's *t*‐test and one‐way analysis of variance (ANOVA) was used to analyze the difference among multiple groups. The Kaplan–Meier method was used to compare differences in mortality rates between groups. The statistical significance was accepted at *p < 0.05, **p < 0.01, and ***p < 0.001.

## Author Contributions

Y.L. conceived the project, designed experiments, and wrote the paper. Z.P. and L.X. supervised the study, designed experiments, performed the experiments, and analyzed the data. S.Z. assisted in data interpretation and edited the manuscript. All authors have read and approved the final manuscript.

## Ethics Statement

The collection and use of the clinical lung cancer tissues was approved by the Ethics Committees of the Shanghai Outdo Biotech Company in accordance with the Declaration of Helsinki (Approval #: YB M‐05‐02). The care and experiments of animals were approved by the Ethics Committees of Central South University (Approval #: CSU‐2022‐0708). The related procedures were in accordance with the Association for Assessment and Accreditation of Laboratory Animal Care guidelines and conformed to the Animal Protection Act of China.

## Conflicts of Interest

The authors declare no conflicts of interest.

## Supporting information



Figure S1. Multiple doses of Gefitinib also fail to sustained induce cytotoxic in human lung cancer cells. A Drug‐stimulating strategy, Human lung cancer cells were stimulated with Gefitinib for seven days. The cell viability was measured daily throughout the experiment. B, C The cell viability of HCC827‐GR (D) and PC9‐GR (E) cell stimulated with Gefitinib (7.5 µM, 15 µM, 22.5 µM) for seven days was measured using CCK‐8 assays (n = 3). B, C Data are shown as mean ± SD. (* *p* < 0.05, ** *p* < 0.01, *** *p* < 0.001, two‐tailed t‐test).
Figure S2. Silencing RIPK1 blocks TAK1 deficiency‐induced PANoptosis in human lung cancer cells with Sepin‐1 and Gefitinib treatment. A, B The cell viability of indicated HCC827‐GR (C) and PC9‐GR (D) cell lines stimulated with or without Gefitinib (7.5 µM), Sepin‐1 (10 µM) and 5z7 (200 nM) for seven days was measured using CCK‐8 assays (n = 3). A, B Data are shown as mean ± SD. (* *p* < 0.05, ** *p* < 0.01, *** *p* < 0.001, two‐tailed t‐test).
Figure S3. PTBP1 mediates Sepin‐1 plus Gefitinib‐induced TAK1 deficiency and PANoptosis formation. A TNIP3, FGFR and PTBP1 mRNA expression determined by real‐time quantitative polymerase chain reaction in human lung cancer cells with or without Gefitinib (7.5 µM) and Sepin‐1 (10 µM) for 24 h (n = 3). B, C The cell viability of indicated HCC827‐GR (C) and PC9‐GR (D) cell lines stimulated with or without Gefitinib (7.5 µM) and Sepin‐1 (10 µM) for seven days was measured using CCK‐8 assays (n = 3). D, E Immunoblot analysis of separase, PTBP1, total RIPK1 (tRIPK1), phosphorylated RIPK1 (pRIPK1), pro‐ (P55) and activated (P30) GSDMD, pro‐ (P55) and activated (P34) GSDME; pro‐ (P35) and cleaved (P17) caspase‐3 (CASP3), pro‐(P55) and cleaved (P18) caspase‐8 (CASP8); total RIPK3 (tRIPK3) and phosphorylated RIPK3 (pRIPK3), total MLKL (tMLKL) and phosphorylated MLKL (pMLKL) in HCC827‐GR‐VECTOR, HCC827‐GR‐PTBP1OE (D), PC9‐GR‐VECTOR and PC9‐GR‐PTBP1OE (E) cell lines after treated with or without Gefitinib (7.5 µM) and Sepin‐1 (10 µM). β‐actin was used as the internal control. A, B, C Data are shown as mean ± SD. (* *p* < 0.05, ** *p* < 0.01, *** *p* < 0.001, two‐tailed t‐test). D and E data are representative of at least three independent experiments.
Table S1.
Table S2.

## Data Availability

The data that support the findings of this study are available in the Supporting Information of this article.
