# Supplementary material for: Separase Inhibition Enhances Gefitinib Sensitivity of Lung Cancer via PTBP1/TAK1/RIPK1‐Mediated PANoptosis
Source: MedComm (2020). 2025 Oct 20;6(11):e70432. doi: 10.1002/mco2.70432 (PMC12536889; doi:10.1002/mco2.70432)
Supplement: Supplementary file 1 — Figure S1. Multiple doses of Gefitinib also fail to sustained induce cytotoxic in human lung cancer cells. A Drug‐stimulating strategy, Human lung cancer cells were stimulated with Gefitinib for seven days. The cell viability was measured daily throughout the experiment. B, C The cell viability of HCC827‐GR (D) and PC9‐GR (E) cell stimulated with Gefitinib (7.5 µM, 15 µM, 22.5 µM) for seven days was measured using CCK‐8 assays (n = 3). B, C Data are shown as mean ± SD. (* p < 0.05, ** p < 0.01, *** p < 0.001, two‐tailed t‐test). Figure S2. Silencing RIPK1 blocks TAK1 deficiency‐induced PANoptosis in human lung cancer cells with Sepin‐1 and Gefitinib treatment. A, B The cell viability of indicated HCC827‐GR (C) and PC9‐GR (D) cell lines stimulated with or without Gefitinib (7.5 µM), Sepin‐1 (10 µM) and 5z7 (200 nM) for seven days was measured using CCK‐8 assays (n = 3). A, B Data are shown as mean ± SD. (* p < 0.05, ** p < 0.01, *** p < 0.001, two‐tailed t‐test). Figure S3. PTBP1 mediates Sepin‐1 plus Gefitinib‐induced TAK1 deficiency and PANoptosis formation. A TNIP3, FGFR and PTBP1 mRNA expression determined by real‐time quantitative polymerase chain reaction in human lung cancer cells with or without Gefitinib (7.5 µM) and Sepin‐1 (10 µM) for 24 h (n = 3). B, C The cell viability of indicated HCC827‐GR (C) and PC9‐GR (D) cell lines stimulated with or without Gefitinib (7.5 µM) and Sepin‐1 (10 µM) for seven days was measured using CCK‐8 assays (n = 3). D, E Immunoblot analysis of separase, PTBP1, total RIPK1 (tRIPK1), phosphorylated RIPK1 (pRIPK1), pro‐ (P55) and activated (P30) GSDMD, pro‐ (P55) and activated (P34) GSDME; pro‐ (P35) and cleaved (P17) caspase‐3 (CASP3), pro‐(P55) and cleaved (P18) caspase‐8 (CASP8); total RIPK3 (tRIPK3) and phosphorylated RIPK3 (pRIPK3), total MLKL (tMLKL) and phosphorylated MLKL (pMLKL) in HCC827‐GR‐VECTOR, HCC827‐GR‐PTBP1OE (D), PC9‐GR‐VECTOR and PC9‐GR‐PTBP1OE (E) cell lines after treated with or without Gefitinib (7.5 µM) and S [file MCO2-6-e70432-s001.pdf]

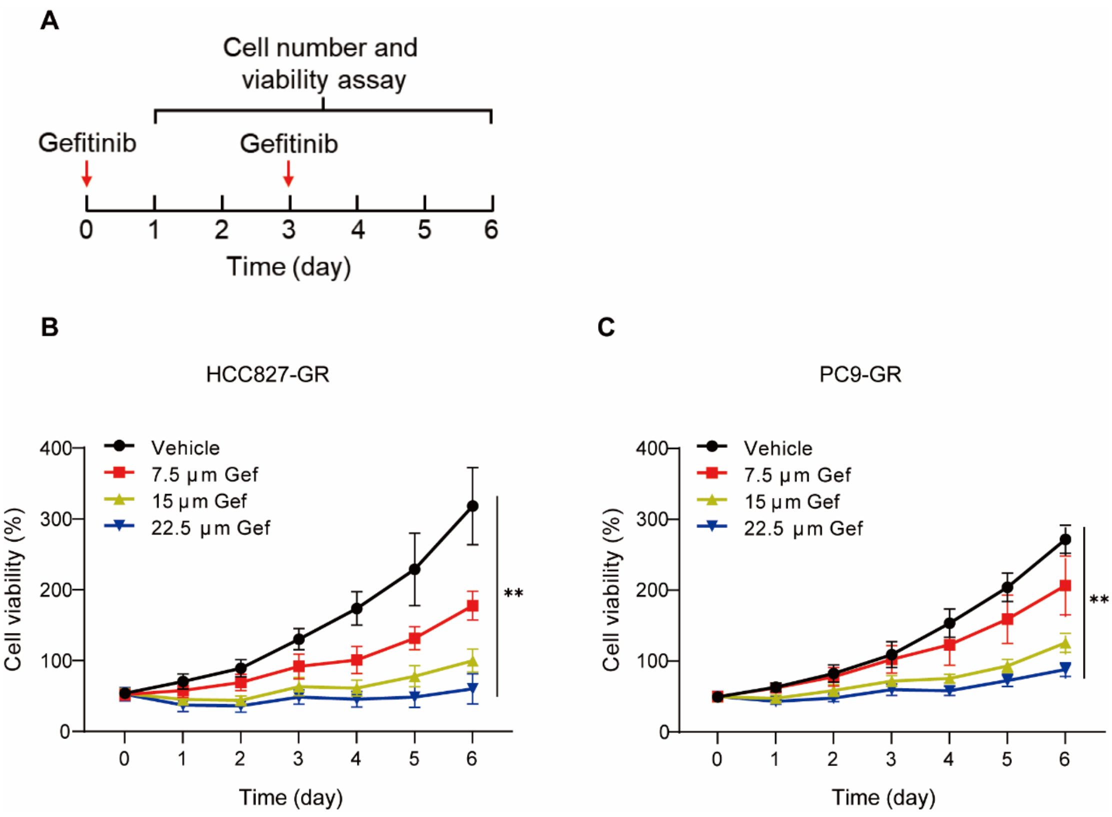

Figure. S1 Multiple doses of Gefitinib also fail to sustained induce cytotoxic in human lung cancer cells.

A Drug-stimulating strategy, Human lung cancer cells were stimulated with Gefitinib for seven days. The cell viability was measured daily throughout the experiment.

B, C The cell viability of HCC827-GR (**D**) and PC9-GR (**E**) cell stimulated with Gefitinib (7.5μM, 15μM, 22.5μM) for seven days was measured using CCK-8 assays (n=3).

B, C Data are shown as mean ± SD. (* $p < 0.05$, ** $p < 0.01$, *** $p < 0.001$, two-tailed t-test).

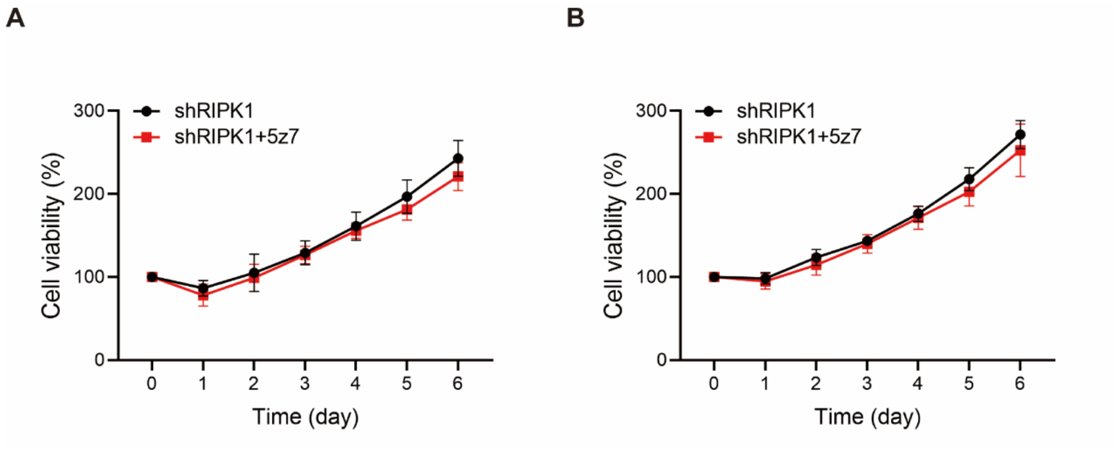

Figure. S2 Silencing RIPK1 blocks TAK1 deficiency-induced PANoptosis in human lung cancer cells with Sepin-1 and Gefitinib treatment.

A, B The cell viability of indicated HCC827-GR (C) and PC9-GR (D) cell lines stimulated with or without Gefitinib (7.5μM), Sepin-1 (10μM) and 5z7 (200nM) for seven days was measured using CCK-8 assays (n=3).

A, B Data are shown as mean ± SD. (* $p < 0.05$, ** $p < 0.01$, *** $p < 0.001$, two-tailed t-test).

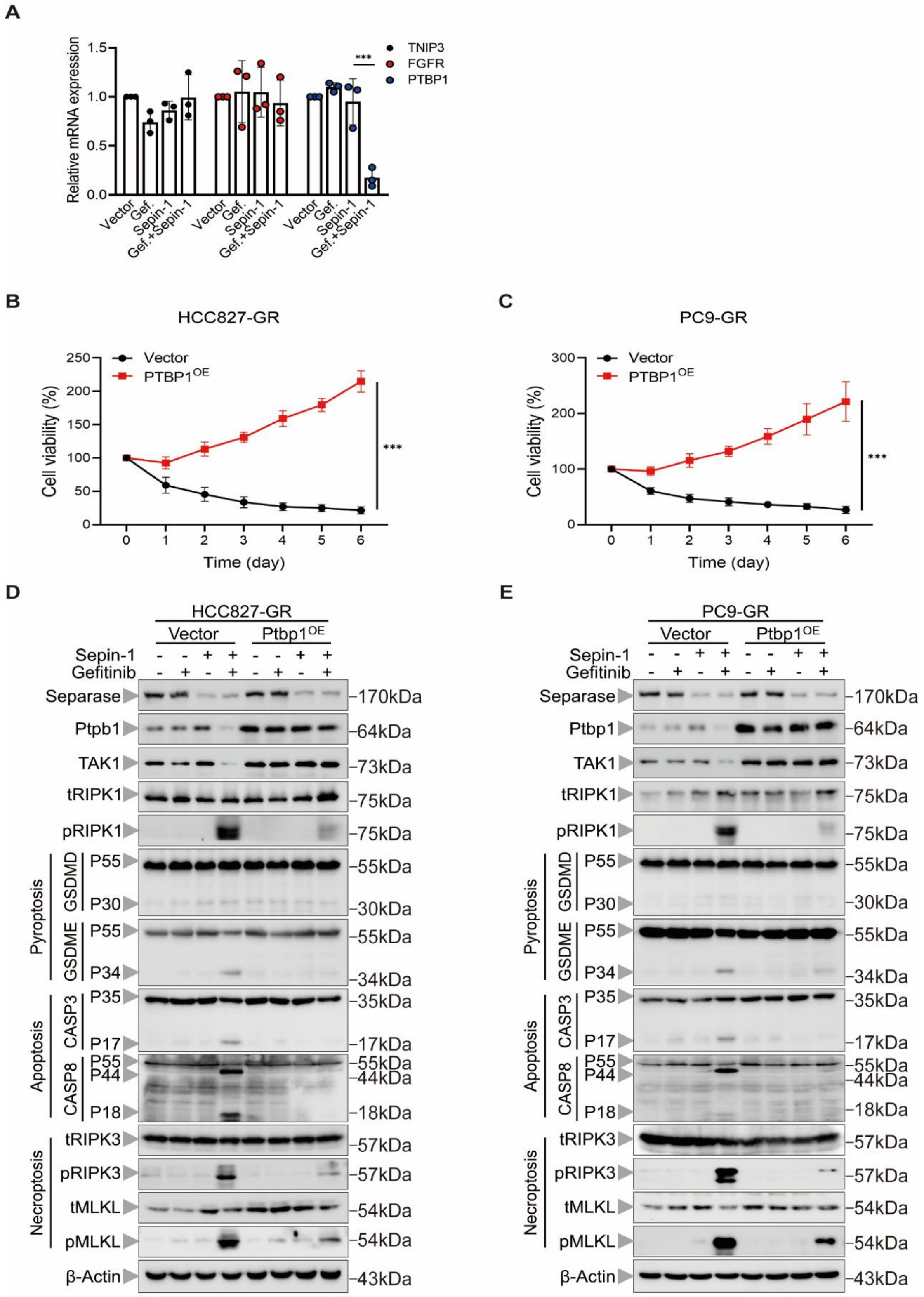

Figure. S3 PTBP1 mediates Sepin-1 plus Gefitinib-induced TAK1 deficiency and PANoptosis formation.

A TNIP3, FGFR and PTBP1 mRNA expression determined by real-time quantitative polymerase chain reaction in human lung cancer cells with or without Gefitinib (7.5μM) and Sepin-1 (10μM) for 24h (n = 3).

B, C The cell viability of indicated HCC827-GR (C) and PC9-GR (D) cell lines stimulated with or without Gefitinib (7.5μM) and Sepin-1 (10μM) for seven days was measured using CCK-8 assays (n=3).

D, E Immunoblot analysis of Separase, PTBP1, total RIPK1 (tRIPK1), phosphorylated RIPK1 (pRIPK1), pro- (P55) and activated (P30) GSDMD, pro- (P55) and activated (P34) GSDME; pro- (P35) and cleaved (P17) caspase-3 (CASP3), pro- (P55) and cleaved (P18) caspase-8 (CASP8); total RIPK3 (tRIPK3) and phosphorylated RIPK3 (pRIPK3), total MLKL (tMLKL) and phosphorylated MLKL (pMLKL) in HCC827-GR-VECTOR, HCC827-GR-PTBP1^{OE} (**D**), PC9-GR-VECTOR and PC9-GR-PTBP1^{OE} (**E**) cell lines after treated with or without Gefitinib (7.5μM) and Sepin-1 (10μM). β-actin was used as the internal control.

A, B, C Data are shown as mean ± SD. (* $p < 0.05$, ** $p < 0.01$, *** $p < 0.001$, two-tailed t-test).

D and E data are representative of at least three independent experiments.

Table. S1

Patient	Position	Histology	Age	Gender	TNM	Stage	Tumor size	Survival time	Death	Recurrence	Separase expression	p-RIPK3 expression	Gefitinib treatment
1	A01	tumor											
	A02	adjacent	77	male	T2aN1M1	2	3.5×3.0×2.5	58	YES	NO	Low	Low	YES
2	A03	tumor											
	A04	adjacent	57	female	T1bN0M0	2	1.5×1.0×1.0	70	NO	NO	Low	High	NO
3	A05	tumor											
	A06	adjacent	73	female	T1aN1M0	2	0.5×0.5×0.5	69	NO	NO	Low	Low	NO
4	A07	tumor											
	A08	adjacent	43	female	T1cN0M0	2	2.5×2.0×1.5	69	NO	NO	Low	High	NO
5	A09	tumor											
	A10	adjacent	61	female	T1cN0M0	2	2.5×2.0×1.5	63	NO	NO	Low	Low	NO
6	A11	tumor											
	A12	adjacent	62	female	T2aN2bM1	2~3	4.0×3.0×2.5	63	YES	YES	Low	Low	NO
7	A13	tumor											
	A14	adjacent	60	male	T2bN0M0	2~3	5.0×4.0×3.0	46	YES	YES	Low	Low	NO
8	A17	tumor											
	A18	adjacent	70	male	T1cN0M1	2~3	2.5×2.0×2.0	56	YES	NO	Low	Low	NO
9	B01	tumor											
	B02	adjacent	69	female	T1cN0M0	2	2.5×1.0×1.0	70	NO	NO	Low	High	NO
10	B03	tumor											
	B04	adjacent	60	female	T1cN1M1	2	3.0×2.0×2.0	37	YES	YES	High	Low	NO
11	B05	tumor											
	B06	adjacent	47	female	T1bN0M0	2	2.0×1.0×1.0	67	NO	NO	Low	High	NO
12	B07	tumor											
	B08	adjacent	71	male	T1bN0M0	2~3	2.0×2.0×2.0	66	NO	YES	Low	Low	NO
13	B09	tumor											
	B10	adjacent	64	male	T1bN0M0	1~2	2.0×2.0×1.5	66	NO	NO	Low	High	NO
14	B11	tumor											
	B12	adjacent	76	male	T2aN0M1	3	4.0×4.0×3.5	43	YES	YES	Low	Low	NO
15	B13	tumor											
	B14	adjacent	68	female	T1cN1M0	2~3	2.4×2.0×1.5	64	NO	YES	Low	Low	NO
16	B15	tumor											
	B16	adjacent	69	male	T2aN0M1	2	4.0×3.0×2.2	64	NO	NO	Low	High	NO
17	B17	tumor											
	B18	adjacent	61	male	T3N0M1	2	6.0×5.0×3.0	43	YES	NO	High	Low	NO
18	C03	tumor											
	C04	adjacent	65	female	T1cN0M0	2	2.5×2.0×1.0	63	NO	NO	Low	Low	NO
19	C05	tumor											
	C06	adjacent	48	male	T1cN0M1	2	3.0×2.0×1.5	38	YES	NO	Low	High	NO
20	C07	tumor											
	C08	adjacent	45	female	T1bN0M0	3	2.0×2.0×1.5	29	YES	YES	Low	Low	NO
21	C09	tumor											
	C10	adjacent	61	female	T1bN1M0	1~2	2.0×2.0×1.52	62	NO	NO	High	Low	NO
22	C11	tumor											
	C12	adjacent	42	female	T1cN2M0	2	3.0×1.8×1.8	62	NO	YES	Low	Low	NO
23	C13	tumor											
	C14	adjacent	53	male	T1cN1M0	2	3.0×2.0×2.0	59	NO	NO	Low	Low	NO
24	C15	tumor											
	C16	adjacent	58	male	T1bN0M0	2~3	2.0×1.5×1.0	59	NO	NO	Low	Low	NO
25	D01	tumor											
	D02	adjacent	60	female	T1aN0M0	1	1.0×0.8×0.5	58	NO	NO	Low	High	NO
26	D03	tumor											
	D04	adjacent	53	male	T1bN1M0	1~2	2.0×1.5×1.0	58	NO	NO	High	Low	NO
27	D05	tumor											
	D06	adjacent	71	male	T1cN2M1	2~3	3.0×2.8×2.5	46	YES	YES	Low	Low	NO
28	D07	tumor											
	D08	adjacent	60	male	T2aN0M0	1	3.5×3.0×2.0	68	NO	NO	Low	High	NO
29	D09	tumor											
	D10	adjacent	71	male	T2bN1M0	2	5.0×3.0×3.0	68	NO	NO	High	Low	NO

30	D11	tumor											
	D12	adjacent	50	female	T1bN0M0	2	2.0×2.0×1.0	44	YES	NO	Low	Low	NO
31	D13	tumor											
	D14	adjacent	52	male	T1bN2M1	2~3	2.0×2.0×1.0	43	YES	YES	Low	Low	NO
32	D15	tumor											
	D16	adjacent	64	male	T1cN0M0	2	3.0×2.5×2.0	67	NO	NO	High	Low	NO
33	D17	tumor											
	D18	adjacent	59	female	T1cN0M0	2	3.0×2.0×2.0	67	NO	NO	Low	Low	NO
34	E01	tumor											
	E02	adjacent	64	female	T4N2M1	2	10.0×8.0×4.0	42	YES	NO	Low	Low	NO
35	E03	tumor											
	E04	adjacent	58	male	T1cN0M0	2	3.0×2.0×2.0	66	NO	NO	Low	High	YES
36	E05	tumor											
	E06	adjacent	62	male	T1cN0M0	2	3.0×2.5×2.0	66	NO	NO	Low	Low	NO
37	E07	tumor											
	E08	adjacent	65	female	T2aN0M0	2	4.0×3.0×3.0	66	NO	NO	High	High	NO
38	E09	tumor											
	E10	adjacent	65	male	T2bN1M0	2	4.5×2.0×2.0	66	NO	NO	High	High	NO
39	E11	tumor											
	E12	adjacent	58	female	T2aN1M0	2~3	3.5×2.0×1.0	65	NO	NO	Low	High	NO
40	E13	tumor											
	E14	adjacent	70	male	T2bN0M0	3	5.0×2.0×2.0	65	NO	NO	Low	High	NO
41	E15	tumor											
	E16	adjacent	79	female	T1bN0M0	2~3	2.0×1.5×1.5	65	NO	NO	Low	High	YES
42	E17	tumor											
	E18	adjacent	52	female	T2bN2M0	1~2	4.5×3.5×2.0	65	NO	YES	Low	High	YES
43	F01	tumor											
	F02	adjacent	60	female	T1cN0M0	2	3.0×3.0×2.5	64	NO	NO	Low	Low	YES
44	F03	tumor											
	F04	adjacent	47	female	T3N2M0	2	7.0×6.0×3.0	41	YES	YES	High	Low	NO
45	F05	tumor											
	F06	adjacent	51	male	T1cN1M0	1~2	3.0×3.0×3.0	43	YES	YES	High	Low	NO
46	F07	tumor											
	F08	adjacent	58	female	T2bN0M0	2	5.0×4.5×3.0	39	NO	NO	Low	Low	NO
47	F09	tumor											
	F10	adjacent	60	male	T1bN0M1	2	2.0×1.5×1.5	52	YES	NO	Low	Low	NO
48	F11	tumor											
	F12	adjacent	71	female	T1cN0M0	2	2.5×2.5×1.5	63	NO	NO	Low	High	NO
49	F13	tumor											
	F14	adjacent	40	female	T2aN0M0	2	3.5×2.5×2.5	62	NO	NO	Low	High	NO
50	F15	tumor											
	F16	adjacent	58	female	T2aN0M1	2	4.0×3.5×3.0	49	YES	NO	Low	Low	NO
51	F17	tumor											
	F18	adjacent	53	female	T1aN2M0	2~3	1.0×0.5×0.5	62	NO	NO	Low	Low	YES
52	G01	tumor											
	G02	adjacent	68	male	T1cN0M0	2	3.0×1.5×1.5	62	NO	NO	High	Low	NO
53	G03	tumor											
	G04	adjacent	63	female	T1bN0M0	1	1.6×1.0×1.0	62	NO	NO	Low	Low	NO
54	G05	tumor											
	G06	adjacent	47	male	T1bN0M0	2	.0~1.2×1.0×0.	61	NO	NO	Low	Low	NO
55	G07	tumor											
	G08	adjacent	72	male	T1cN0M1	2~3	3.0×1.5×1.5	37	YES	NO	Low	Low	NO
56	G09	tumor											
	G10	adjacent	40	female	T1cN1M0	2	3.0×2.5×2.0	34	YES	YES	High	Low	NO
57	G11	tumor											
	G12	adjacent	77	male	T1cN0M1	2	2.7×1.5×1.5	14	YES	NO	Low	Low	YES
58	G13	tumor											
	G14	adjacent	65	female	T1cN1M0	2	2.5×1.5×1.5	36	NO	NO	Low	Low	NO
59	G15	tumor											
	G16	adjacent	68	male	T1bN0M0	1	2.0×1.5×0.5	30	NO	NO	Low	High	NO
60	G17	tumor											
	G18	adjacent	77	female	T1cN0M0	1	2.5×2.0×1.5	20	YES	YES	High	Low	NO

61	H01	tumor												
	H02	adjacent	51	male	T2aN0M0	2	4.0×3.5×3.5	29	YES	YES	High	Low	YES
62	H03	tumor												
	H04	adjacent	64	male	T1cN1M1	2	2.5×1.5×1.5	32	YES	NO	Low	High	NO
63	H05	tumor												
	H06	adjacent	59	male	T1bN0M1	1~2	2.0×2.0×2.0	38	YES	NO	High	Low	NO
64	H07	tumor												
	H08	adjacent	65	male	T1cN0M0	2	2.5×2.0×1.5	36	YES	YES	High	High	NO
65	H09	tumor												
	H10	adjacent	66	male	T1cN1M1	2	2.5×2.0×1.0	33	YES	NO	Low	Low	NO
66	H11	tumor												
	H12	adjacent	45	male	T1aN2M1	2~3	1.0×0.5×0.5	55	YES	NO	Low	Low	NO
67	H15	tumor												
	H16	adjacent	52	male	T1bN0M0	2	1.2×0.5×0.5	34	YES	YES	Low	Low	NO
68	H17	tumor												
	H18	adjacent	61	male	T1bN0M0	3	1.7×1.5×1.5	34	YES	NO	Low	Low	NO
69	I01	tumor												
	I02	adjacent	52	male	T1cN0M0	2	2.5×2.0×1.5	33	NO	NO	Low	Low	NO
70	I03	tumor												
	I04	adjacent	73	male	T1bN0M1	1	2.0×2.0×1.8	34	YES	NO	High	High	NO
71	I05	tumor												
	I06	adjacent	42	male	T1cN2M0	2	2.5×2.0×2.0	33	YES	YES	High	Low	NO
72	I07	tumor												
	I08	adjacent	66	female	T1cN1M1	2	3.0×2.0×2.0	34	YES	NO	High	Low	NO
73	I09	tumor												
	I10	adjacent	64	female	T1bN0M0	2	2.0×1.5×0.4	32	NO	NO	High	Low	YES
74	I11	tumor												
	I12	adjacent	59	female	T1cN2M1	2	2.2×2.0×1.5	34	YES	NO	High	Low	YES
75	I15	tumor												
	I16	adjacent	43	female	T1cN1M0	2~3	3.0×2.0×1.0	58	NO	NO	High	Low	NO
76	I17	tumor												
	I18	adjacent	56	female	T2aN1M1	2	3.5×3.5×3.3	58	YES	YES	High	Low	NO
77	J01	tumor												
	J02	adjacent	59	female	T1bN0M0	1~2	2.0×2.0×1.0	67	NO	NO	Low	High	NO
78	J03	tumor												
	J04	adjacent	63	female	T1bN1M0	2	2.0×2.0×1.0	64	NO	NO	Low	Low	NO
79	J05	tumor												
	J06	adjacent	64	male	T2aN0M0	1	4.0×3.0×3.0	58	NO	NO	Low	High	YES
80	J07	tumor												
	J08	adjacent	65	male	T1cN1M0	3	2.7×1.5×1.5	62	NO	NO	Low	Low	NO
81	J09	tumor												
	J10	adjacent	49	male	T3N0M1	2	7.0×4.0×3.0	50	YES	NO	Low	Low	NO
82	J11	tumor												
	J12	adjacent	61	male	T2aN1M0	2	3.5×2.0×2.0	35	YES	YES	High	Low	NO
83	J13	tumor												
	J14	adjacent	46	female	T1cN0M1	2	2.5×1.5×1.5	35	YES	NO	Low	High	YES
84	J15	tumor												
	J16	adjacent	52	male	T1cN0M0	2	3.0×3.0×3.0	65	NO	NO	High	Low	NO
85	J17	tumor												
	J18	adjacent	50	male	T2aN2M1	3	4.0×3.0×3.0	50	YES	YES	Low	Low	NO

Table. S2

No.	Name		No.	Name		No.	Name
1	Sepin-1		21	IDO-IN-2		41	Deucravacitinib
2	Cobimetinib		22	Navoximod		42	Cediranib
3	Pictilisib		23	Belvarafenib		43	Orelabrutinib
4	Vismodegib		24	ARS-853		44	Bemcentinib
5	AR-00341677		25	Etomoxir		45	Sulfopin
6	Apitolisib		26	Tenalisib		46	Pinometostat
7	Ipatasertib		27	Avitinib		47	Silodosin
8	RG-7603		28	Glumetinib		48	Zolmitriptan
9	Taselisib		29	Selonsertib		49	Bozitinib
10	GDC-0152		30	Naquotinib		50	Imaradenant
11	Ravoxertinib		31	Tofogliflozin		51	Duvelisib
12	G-868		32	Evobrutinib		52	Mivebresib
13	Paxalisib		33	Upadacitinib		53	Volinanserin
14	GDC-0326		34	Pizuglanstat		54	Remibrutinib
15	Brilanestrant		35	Zanubrutinib		55	Olodanrigan
16	GDC-0575		36	Verdiperstat		56	Tubacin
17	Fenebrutinib		37	Tegoprazan		57	Varenicline
18	Inavolisib		38	Norgestimate		58	Larotrectinib
19	Divarasib		39	Irosustat		59	Selitrectinib
20	Venetoclax		40	Gemigliptin		60	Pirtobrutinib
